# Multi-scale high-throughput phenotyping of apple architectural and functional traits in orchard reveals genotypic variability under contrasted watering regimes

**DOI:** 10.1038/s41438-019-0137-3

**Published:** 2019-05-01

**Authors:** Aude Coupel-Ledru, Benoît Pallas, Magalie Delalande, Frédéric Boudon, Emma Carrié, Sébastien Martinez, Jean-Luc Regnard, Evelyne Costes

**Affiliations:** 1 UMR AGAP, Univ Montpellier, CIRAD, INRA, Montpellier SupAgro, 34398 Montpellier Cedex 5, France; 2 CIRAD, 34398 Montpellier Cedex 5, France; 3 Present Address: University of Bristol, School of Biological Sciences, Life Science Building, 24 Tyndall Avenue, Bristol, BS8 1TQ UK

**Keywords:** High-throughput screening, Plant stress responses

## Abstract

Despite previous reports on the genotypic variation of architectural and functional traits in fruit trees, phenotyping large populations in the field remains challenging. In this study, we used high-throughput phenotyping methods on an apple tree core-collection (1000 individuals) grown under contrasted watering regimes. First, architectural phenotyping was achieved using T-LiDAR scans for estimating convex and alpha hull volumes and the silhouette to total leaf area ratio (*STAR*). Second, a semi-empirical index (*I*_PL_) was computed from chlorophyll fluorescence measurements, as a proxy for leaf photosynthesis. Last, thermal infrared and multispectral airborne imaging was used for computing canopy temperature variations, water deficit, and vegetation indices. All traits estimated by these methods were compared to low-throughput in planta measurements. Vegetation indices and alpha hull volumes were significantly correlated with tree leaf area and trunk cross sectional area, while *I*_PL_ values showed strong correlations with photosynthesis measurements collected on an independent leaf dataset. By contrast, correlations between stomatal conductance and canopy temperature estimated from airborne images were lower, emphasizing discrepancies across measurement scales. High heritability values were obtained for almost all the traits except leaf photosynthesis, likely due to large intra-tree variation. Genotypic means were used in a clustering procedure that defined six classes of architectural and functional combinations. Differences between groups showed several combinations between architectural and functional traits, suggesting independent genetic controls. This study demonstrates the feasibility and relevance of combining multi-scale high-throughput methods and paves the way to explore the genetic bases of architectural and functional variations in woody crops in field conditions.

## High-throughput phenotyping for plants

A high-throughput method for determining plant characteristics offers a viable way to track competitive traits in the field. Increasingly difficult agricultural conditions are spurring plant breeding programs to prioritize plants with strong adaptations for healthy metabolism, but no current technologies can rapidly assess plant traits in the field. Now, Benoît Pallas, of the French National Institute for Agricultural Research, and his team have adapted tools such as infrared and laser-light scanning, and drone imagery, to take define and correlate metrics of 1000 apple trees, such as measurements of volume, leaf area, photosynthetic activity, and water usage. With these results and prior knowledge of the trees’ genotype, the team was able to group the trees into six classes with distinct physical and functional trait combinations. These techniques may open doors for discovering the genetic bases of plant traits.

## Introduction

The biophysical approach proposed by Monteith (1977)^1^ states that plant production potential relies on traits associated with plant capacity to intercept solar radiation, to convert this energy into carbohydrates and allocate them to the harvested fruits or seeds. In fruit trees, breeding programs did not account for these elementary traits and rather focused on disease resistance, fruit organoleptic properties, and yield^2^. Moreover, in the current climatic context characterized by an increase in temperature and a risk of water scarcity, targeting new varieties with high performance even under constraining conditions becomes crucial for breeding programs. Potted experiments in phenotyping platforms have arisen as performing approaches to phenotype perennial species^3^. These platforms have led to encouraging results in young potted trees through the identification of large genotypic variability in architectural (plant height, total leaf area) and functional (transpiration, water-use efficiency) traits^4^. These studies were made possible by using new technologies provided in those platforms^5^, i.e., Red Green Blue (RGB) images for plant growth estimation, and automated plant transpiration measurements. Nevertheless, such technologies are not available in field conditions and new developments are necessary to enable the phenotyping of both architectural and functional traits of woody crops at different developmental stages, including adult and fruiting.

Plant architecture determines many traits associated with plant performance such as flowering intensity and intra-tree location, or light interception efficiency. Most of the studies that have dealt with genetic variations of architectural traits have focused on simple plant traits, such as height or trunk diameter^6^, whose measurements are feasible on large populations. More local traits, such as maximal internode length along trees, branching density or mean axis length were also considered on young trees^7^. These studies have shown that architectural traits are genetically controlled, and identified quantitative trait loci (QTL) associated with their variations although these traits display high polygenetic determinisms. Nevertheless, other properties of tree canopies such as the efficiency of architectures for intercepting solar radiation or for optimizing intra-canopy microclimate (temperature, humidity) could be relevant for describing between-trees variability. High-throughput (HT) estimations of traits such as the silhouette to total leaf area ratio (*STAR*^8^) or the leaf density within the tree canopy could help characterizing genotype efficiencies. Methods based on three-dimensional (3D) digitizing were successfully used for characterizing whole tree architecture^8^ but they remain time-consuming and not compatible with HT phenotyping. Terrestrial LiDAR (T-LiDAR) scanners have recently emerged as promising tools for measuring 3D vegetation structures. T-LiDAR scans have been used in many studies, mainly in forest but also in agronomic contexts^9^, notably for evaluating individual crown structure^10^ or leaf area density^11^. More recently, a study reported alpha hull volume estimation based on merging RGB images of tree orchards for assessing tree space occupation^12^. However, to the best of our knowledge, T-LiDAR has never been deployed in a context of HT phenotyping for genetic studies.

Measurements of carbon and water-related processes in plants are frequently carried out with InfraRed Gas-Analyzers (IRGAs). These devices allow precise assessment of leaf functions, but are time-consuming. Other variables, associated with chlorophyll fluorescence have been proposed to quickly estimate leaf photosynthesis through direct measurements on leaves or by chlorophyll fluorescence emission imaging^13^. The most used variables were the effective quantum efficiency of photosystem II (PSII), the derived electron transport rate and the photochemical and non-photochemical quenching coefficients^13,14^. These parameters have been used in genetic studies in which associated QTLs have been detected^15^. Nevertheless, variation in the relationship between these variables and photosynthetic rate has been observed, mainly under water stress due to complex regulation of stomatal closure and subsequent repercussions on leaf fluorescence^16^. Losciale et al.^17^. recently proposed a fast method combining measurements, without dark adaptation, of chlorophyll fluorescence, carboxylative activity of the RuBisCo and temperature-based variables. The authors have shown that a resulting variable, the *I*_PL_ index, was strongly and linearly correlated to net photosynthesis in apple and pear under contrasted soil water supply. The *I*_PL_ thus appears as a promising indicator, but has never been tested at HT.

Remotely sensed imagery in field crops have received large interest for rapid estimation of yield or responses to soil water deficit^18^. Recently, both multispectral (MS) and thermal infrared (TIR) imaging have been used for phenotyping large populations of individuals in annual species and performing genetic analyses on the estimated traits^19,20^. However, the related developments have mainly been tackled toward homogeneous plant cover and are not fully adapted to non-fully covering crops, like orchards. Analytical adaptations were developed in apple tree to estimate water stress indicators at the tree scale within biparental populations^21^, on which genetics analyses^22^ were performed. In these studies, the impact of water stress was computed based on the water deficit index (*WDI*) for partially soil covering vegetation. Although promising, these studies did not estimate other indicators provided by MS imaging. Among others, *NDVI*^23^ (Table 1) is considered as a proxy for leaf area index, and intercepted solar radiation; *GNDVI*^24^ and *MCARI2*^25^ are quite similar to *NDVI* but display more (*GNDVI*) or less (*MCARI2*) sensitivity to chlorophyll concentration; while *PRI*^26^ accounts for the state of PSII and is considered as a proxy for radiation-use efficiency.

**Table 1:** Description of the variables used in the study, with their respective methods of measurement and units

Trait abbreviations	Definition	Unit	Method
*TCSA*	Trunk cross sectional area	cm²	Manual measurements
*TLA*	Tree leaf area	m²	
*c_volume*	Volume of the 3D convex hull	m^3^	T-LiDAR
*a_volume*	Volume of the 3D alpha hull	m^3^	
*c*_i_	Convexity index		
*STAR*	Silhouette to total leaf area ratio		
*NDVI*	Normalized difference vegetation index		Multispectral imaging
*GNDVI*	Green NDVI		
*MCARI2*	Modified chlorophyll absorption ratio index improved		
*PRI*	Photochemical reflectance index		
*WDI*	Water deficit index		
*pix_num*	Vegetation pixel number		
*T*_surf_ *–T*_air_	Difference between canopy surface and air temperatures	°C	Thermal infrared imaging
*A*_n_	Net photo-assimilation	µmol m^−2 ^s^−1^	Infrared gas-analyzer (IRGA)
*I*_PL_	Photo-assimilation performance index	µmol m^−2^ s^−1^	IRGA with fluorimeter
*T*_leaf_ -*T*_air_	Difference between leaf surface and air temperature	°C	
*P*_KO/KC_	Computed leaf fluorescence parameter	µmol m^−2^ s^−1^	
*g*_s_	Stomatal conductance	mmol m^−2^ s^−1^	Porometer

In this article, we describe for the first time in a woody plant in an orchard, the deployment of in field multi-scale and multi-objectives HT measurements on a core-collection of 241 genotypes representative of the European diversity of apple tree^27^. Fluorescence, airborne imagery and T-LiDAR techniques were deployed on the whole core-collection, and complemented by fine, *in planta* characterization (stomatal conductance, photosynthesis, leaf area) on a subset of contrasted genotypes. Effects of the genotype and watering scenario were dissected and a wide genotypic variability was identified for all traits. A clustering method was then used for determining genotype classes based on combinations of architectural and functional traits.

## Material and methods

### Plant material and experimental set-up

This study was carried out on an apple tree core-collection^27^ previously characterized for architectural and functional traits at 1-year-old stage into a HT phenotyping platform in controlled conditions^4^. The 241 genotypes of the core-collection were re-multiplied at INRA Angers and grafted onto M9 Pajam® 2 rootstock before being planted in field in February 2014 at the INRA experimental unit ‘DiaScope’’ in Montpellier, France (43°36 N, 03°58E). Trees were planted at 5 × 2 m distances, irrigated using micro-sprayers located between-trees, and left unpruned. Trees were not thinned from planting until 2016. In 2017, trees were manually thinned in mid-June to maintain one fruit per inflorescence. The orchard (1.2 ha) comprised ten rows of 100 trees, embedding two replications of two trees per genotype randomly distributed within the field. Well-watered (WW) and water deficit (WD) tree rows alternated within the trial, one WW tree facing one WD for each genotype. During the two first years after planting, no limiting watering regime was implemented to ensure a proper development of all trees. Differential watering scenarios were set-up on WW and WD lines in 2016 and 2017 and consisted in withholding irrigation on WD lines for one month during summer. Soil water potential (*Ψ*_soil_) was monitored by Watermark® tensiometric probes (−30 and −60 cm depth) connected to Agribase® dataloggers on a subset of cultivars having shown contrasted vigor in a previous greenhouse experiment^4^. Results reported here are focused on the 2017 experimental campaign only. That year, the mean value of harvest fruit weight per tree over the population was 8.43 kg with a maximum of 39.48 kg. Although this 4^th^ year after planting corresponded to the first year of significant fruit production for most trees, some of them (8%) did not bear any fruit. From 7^th^ July to 2^nd^ August 2017, WW trees received 2 h irrigation per day, while WD trees were watered 2 h per day twice a week only. The water deficit was gradually established after irrigation withholding and *Ψ*_soil_ reached up to –120 kPa for WD trees at the end of this period (late July 2017, Supplementary Fig. S1) while *Ψ*_soil_ on WW trees was maintained close to 0 kPa. All measurements presented hereafter were carried out at the end of July 2017, when contrast in *Ψ*_soil_ between WW and WD trees nearly reached its maximum, except for the T-LiDAR acquisition, which was carried out in early October 2017.

### Thermal infrared and multispectral imaging

#### Image acquisitions and experimental set-up

Airborne imaging acquisition was carried out on clear sunny days, on 27^th^ July from 1 to 2 p.m. and on 28^th^ July from 9:30 to 10:30 a.m. (UTC). The vector was a Mikrokopter® hexa-rotor drone (www.phenome-fppn.fr/phenome_eng/Facilities/Montpellier-Field) operating at 25 m height. Three successive elevations were performed each day to cover the entire field, hence around 45 min were needed between the first and last image acquisition in the field. Zenithal images were acquired at frequency allowing image along-track and across-track overlaps of 80% and 70%, respectively. The on-ground field set-up comprised (i) gray targets for radiometric correction, (ii) contrasted thermal targets (2 m²) used during the flights and measured by IR120 thermoradiometers (Campbell Sci.), (iii) ground control points GPS-RTK (Global Position System–Real Time Kinematic) geo-referenced (2.5 cm accuracy), and (iv) a meteorological station acquiring air temperature and relative humidity, wind speed and direction, and global radiation at 10 s time-step. TIR and MS images were acquired, respectively, by a FLIR® TAU2.7 uncooled camera (7.5–13 µm, resolution of 640 × 512 pixels) and an AirPhen v3 camera (www.hiphen-plant.com) measuring reflectance in blue (450 nm), green (530 and 570 nm), red (675 nm), red-edge (730 nm), and NIR (850 nm) with a bandwidth close to 10 nm.

#### Vegetation and water stress index computation

Data post-processing (radiometric correction, image ortho-rectification, geolocation and mosaicking) was performed with Erdas Imagine Pro, ExifTool and Agisoft Photoscan softwares. Linear regressions between contrasted targets and IR120 temperatures allowed transforming the numerical values of image pixels into temperatures^28^. GPS coordinates were used to accurately localize each single tree of the experiment and a 0.70 m radius zone (buffer zone, Supplementary Fig. S2) was delineated around tree centers. For the canopy temperature (*T*_surf_) calculation, the leaf MS signature was used to classify images and discard soil pixels. Differences between surface and air temperatures (*T*_surf _–*T*_air_)^29^ were then computed by subtracting the mean air temperature during the acquisition. MS-based index values (*NDVI*, *GNDVI*, *PRI*, and *MCARI2*) were extracted for each pixel within the buffer zone (Supplementary Table S1). For each index, mean and standard deviation of pixels values were calculated inside the buffer zone, considering vegetation pixels for *T*_surf _*–* *T*_air_ or all the pixels for MS-based indices. The *WDI* (Supplementary Table S1) was computed based on a trapezoid shape built from the scatterplot between *T*_surf_ *–* *T*_air_ and *NDVI*^21,30^. *WDI* is an indicator used for estimating the effect of water deficit on leaf transpiration, varying from 0 (maximal transpiration) to 1 (no transpiration).

### High-throughput estimation of leaf photosynthesis

#### Estimation of a semi-empirical index (*I*_PL_)

Following Losciale et al.^17^, the semi-empirical index *I*_PL_ was used as a proxy for net leaf photosynthetic rate (*A*_*n*_). It was computed as a linear combination of the electron transport rate exiting from PSII (*J*_PSII_, derived from the effective PSII quantum efficiency, *ΦPSII*) aggregated with the Michaelis–Menten constants for carboxylation (*K*_C_) and photorespiration (*K*_O_) forming a new variable, *P*_KO_/K_C_ = *J*_PSII_ × *K*_O_/*K*_C_, and the leaf-to-air temperature difference (*T*_leaf_ – *T*_air_). Calibration of the linear regression was achieved through measurements on a subset of six genotypes (24 trees). Leaf gas-exchanges were measured using an open circuit InfRared Gas-exchange Analyzer (IRGA) fitted with a leaf fluorimeter and a light-emitting diode light source (LI-6400, LI-COR Inc., Lincoln, NE, USA) on 30^th^ July. Measurements were performed with two LI-COR devices on fully expanded, fully exposed leaves and repeated at three time-periods: morning, midday, afternoon with actinic light set to 1500 μmol m^−2^ s^−1^. During each measurement, we recorded *A*_*n*_, stomatal conductance (*g*_s_), leaf (*T*_leaf_) and air (*T*_air_) temperatures, maximum fluorescence with light-adapted leaf (*F*_m_’),  and steady state fluorescence (*F*_s_) in order to compute *ΦPSII*. We collected with each device, respectively, 98 (LI-COR 1) and 132 (LI-COR 2) measurements. In the calibration process, two thirds of the data were used for estimating model coefficients in the two datasets. For each device, linear models were established gathering all the data collected, and their validity was assessed across watering scenarios or time of the day. Model validation was performed on the remaining third of the datasets, using the coefficients determined in the calibration process.

#### High-throughput measurements of *I*_PL_

HT measurements were performed on 4 trees × 195 genotypes during 2 consecutive days (27^th^ and 28^th^ July) with the two LI-COR devices complementarily. Measurements consisted in rapid (ca. 30 s per leaf) acquisition of *ΦPSII*, *T*_leaf_, and *T*_air_, performed during a maximum period of 3 h around solar midday. Parameters calculated in the calibration process were then used for each LI-COR to calculate *I*_PL_ values.

#### In planta measurement of leaf stomatal conductance

Stomatal conductance (*g*_s_) was measured on 27^th^ July on a subset of eight genotypes (two WW and two WD trees per genotype) with a porometer (AP4; Delta-T Devices Ltd). Measurements were performed on three fully expanded, fully sun-exposed leaves per tree, during a period of 1 h 30 min centered around solar midday. Stomatal conductance values were then compared to *T*_surf_ – *T*_air_ and *WDI* estimated from TIR imaging and to *T*_leaf_ – *T*_air_ estimated with IRGAs  to assess the ability of the difference between air and vegetation temperatures to account for functional differences.

### High-throughput phenotyping of whole-tree leaf area and light interception

#### T-LiDAR data acquisition

**Fig. 1:**
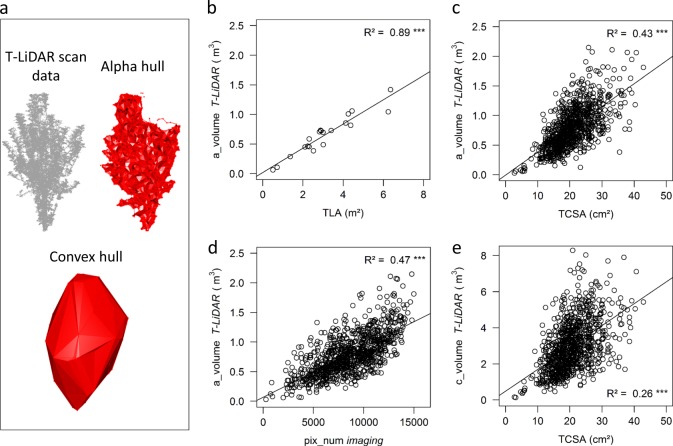
Representation of T-LiDAR variables, and comparisons with imaging data and direct plant measurements. **a** Example of three-dimensional (3D) alpha and convex hulls computed on one 4-years-old tree scanned with T-LiDAR, in autumn 2017. **b** Correlation between alpha hull volume (*a_volume*) and total leaf area (*TLA*) computed on a subset of 20 trees in autumn 2017. **c** Correlation between *a_volume* and trunk cross sectional area (*TCSA*) measured on the whole core-collection in autumn 2017 (*n* = 930 trees). **d** Correlation between *a_volume* and vegetation pixel numbers (*pix_num*) in the airborne image buffer zone, on the whole core-collection. **e** Correlation between convex hull volume (*c_volume*) and *TCSA*. Coefficients of determination and their significance are indicated in each panel

Data acquisition was performed on the whole population in October 2017 after harvest, with a RIEGL VZ400 terrestrial laser scanner (RIEGL Laser Measurement Systems GmbH, Horn, Austria). Scans were carried out with a 360° view each ten meters on every row with an angular resolution of 0.04°. Scans were then registered in a common coordinates system with the Riegl software (RiSCAN PRO v2.0.1) and point clouds were processed using the Computree platform (http://computree.onf.fr/) (Fig. 1a) to remove noise and outlier points.

#### Plant shape and light interception descriptors

Volumes of convex hulls (*c_volume*) of the tree point clouds were computed using the PlantGL library^31^ within OpenAlea platform^32^ and volumes of alpha hulls (*a_volume*) with R software^33^ using the alphashape3d library. The *c_volume* reflects the maximal space occupation of the tree, while *a_volume* is an extension of the convex hull allowing the creation of concave envelopes around the point cloud and is consequently more associated with the space really occupied by the trees. Creation of concavities in the alpha hull depends on a parameter (*α*) whose value was estimated on a subset of 20 trees on which the total tree leaf areas (*TLAs*) were estimated by randomly collecting one leaf over five within the canopy. Individual areas of collected leaves were then measured using a leaf area meter (LI-COR 3100, Area Meter; Lincoln, NE, USA). The chosen *α* value (0.15) was the one maximizing the correlation between *a_volume* and *TLA* on this subset of trees. A convexity index (*c*_i_) was computed as the ratio of *a_volume* to *c_volume* to evaluate the density of tree space occupation within its convex hull. A proxy of light interception efficiency, the *STAR*, was computed in PlantGL following previously published methods^34^.

The trunk cross sectional area (*TCSA*) was computed from the trunk circumference acquired with a tape measure on the whole core-collection in September 2017. As *TCSA* is known as a relevant indicator of plant vigor^35^, it was directly compared to the canopy volumes (T-LiDAR) and to the vegetation indexes (MS imaging).

### Statistical analyses

Analyses were performed using R software^33^. For each trait, models were selected based on the lowest Bayesian Information Criterion, among several mixed-effect models (Supplementary Table S2). Models always included a random genotypic effect and a fixed-effect of the scenario, completed or not by other fixed effects (line, daytime period, gas-exchange device, climatic conditions). The best linear unbiased predictors (BLUPs) of genetic values were estimated for each trait with the selected model. Variance components were then used to estimate the broad-sense heritability (*H*^2^) as:

1 $$H^2 = \frac{{\sigma _G^2}}{{\left[ {\sigma _G^2 + \frac{{\sigma _R^2}}{n}} \right]}}$$

with ${\sigma _{\rm{G}}^2}$ the genetic variance, ${\sigma _{\rm {R}}^2}$ the residual variance, *n* the number of replicates per genotype.

Multivariate analyses were performed on the  genetic values. Pearson coefficients of correlation between variables were evaluated, considering phenotypic and genotypic values. A PCA and a Hierarchical Ascendant Classification based on Ward method were performed, using variables related to architectural and functional traits and estimated from the three different HT methods. Some variables closely correlated to other ones were not considered in these multivariate analyses to limit redundancies.

## Results

### Characterization of tree architecture and effect on light interception efficiency

Correlation between *a_volume* and *TLA* (Table 1), assessed on 20 trees with *TLA* ranging from 0.3 to 6.2 m², was highly significant (R² = 0.89, Fig. 1b). *a_volume* was positively, highly significantly correlated to *TCSA* on the whole core-collection (Fig. 1c, *R*² = 0.43). *a_volume* was also highly correlated to *pix_num* estimated from the zenithal airborne images (Fig. 1d, *R*² = 0.47). By contrast, the correlation between *c_volume* and other variables related to tree vigor were lower (*R*² = 0.26, for both *TCSA* and *pix_num*, Fig. 1e and Supplementary Fig. S3).

All parameters estimated from T-LiDAR exhibited strong effect of the genotype and high heritability values (*H*² from 0.77 to 0.86; Table 2). A slight but significant effect of the watering scenario was found on all traits (except *a_volume*), associated with a moderate decrease in the size of the trees under WD as compared to WW conditions (e.g., –3.7% and –10.4% for *a_volume* and *c_volume*, respectively). Higher *c*_i_ was observed under WD, suggesting a higher leaf area density. Consistently, higher leaf area density led to WD trees with lower *STAR* values (Table 2).

**Table 2:** Range of variability for the traits (mean ± SD) measured on the core-collection of apple trees, with effects of the genotype, of the watering scenario, and broad-sense heritability

Traits	Mean±SD		*P*_G_	*P*_S_	*P*_*D*_	*H*²
	WW	WD				
*TCSA* (cm^2^)	20.7 ± 0.47	19.6 ± 0.45	***	***	–	0.73
*c_volume* (m^3^)	3.17 ± 1.44	2.84 ± 1.32	***	***	–	0.82
*a_volume* (m^3^)	0.81 ± 0.36	0.78 ± 0.33	***	ns	–	0.77
*c*_i_	0.27 ± 0.09	0.30 ± 0.10	***	***	–	0.86
*STAR*	0.75 ± 0.15	0.71 ± 0.14	***	***	–	0.78
*NDVI*	0.34 ± 0.09	0.29 ± 0.09	***	***	***	0.85
*GNDVI*	0.23 ± 0.09	0.24 ± 0.10	***	ns	***	0.79
*MCARI2*	0.55 ± 0.13	0.43 ± 0.14	***	***	ns	0.87
*PRI*	−0.048 ± 0.049	−0.061 ± 0.042	***	***	***	0.69
*WDI*	0.18 ± 0.18	0.30 ± 0.08	***	***	***	0.70
*pix_num*	9582 ± 2512	8466 ± 2786	***	***	***	0.84
*T*_surf _– *T*_air_ (°C)	0.82 ± 2.15	2.88 ± 2.21	***	***	***	0.63
*I*_PL_	14.0 ± 5.93	9.72 ± 5.61	***	***	–	0.43

Acronyms as in Table 1. The significance of genotype (*P*_G_), watering scenario (*P*_S_), and date effects (*P*_D_) was tested considering a linear fixed-effect model without interaction. ****p ≤ 0.001; ns, non-significant. Date effect is considered for imaging data, only

**Fig. 2:**
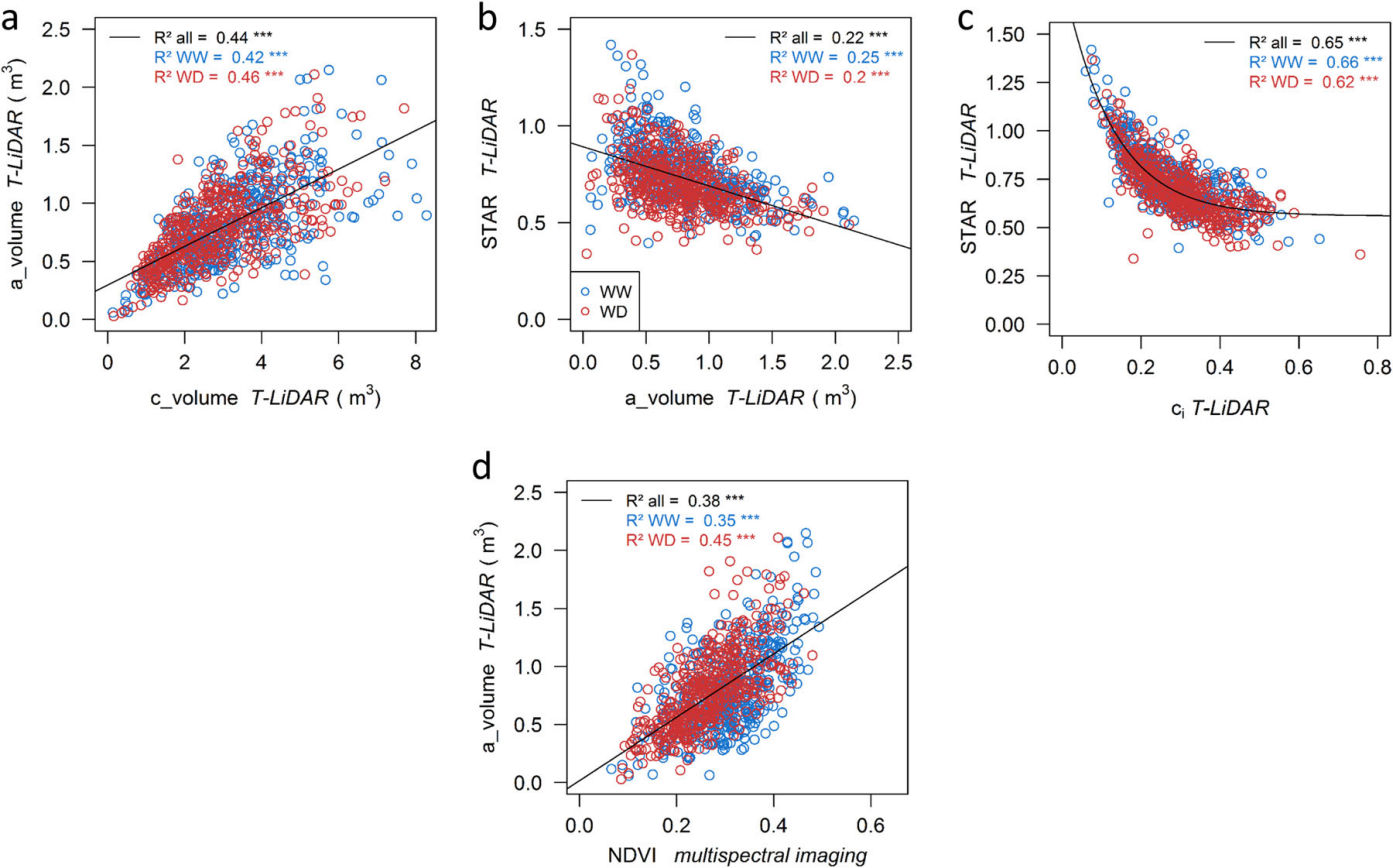
Correlations between T-LiDAR and multispectral (MS) imaging indices. **a**–**c** Correlations between T-LiDAR variables on the whole core-collection (*n* = 930 trees): alpha hull (*a_volume*) and convex hull volumes *(c_volume)*, silhouette to total leaf area ratio (*STAR*) and convexity index (*c*_i_). **d** Correlation between *a_volume* and *NDVI* estimated from MS imaging. Blue and red points represent trees subjected to well-watered (WW) or water deficit (WD) conditions, respectively. For each correlation, the coefficient of determination and its significance was computed considering either the whole dataset (all), or each watering scenario independently (WW or WD)

We explored the correlations between the variables derived from T-LiDAR measurements to evaluate the relationship between tree vegetative architecture and light interception efficiency as estimated by *STAR* values. *a_volume* was positively correlated to *c_volume* whatever the watering scenario with *R*² = 0.44 (Fig. 2a) showing however a range of contrasted combinations of *a_volume* and *c_volume* values within the core-collection. *STAR* was negatively correlated with *c*_i_ (Fig. 2c; *R*² = 0.65, power function) and weakly, negatively correlated with *a_volume* (Fig. 2b; *R*² = 0.22). Finally, *a_volume* was significantly, but not tightly, correlated to *NDVI* estimated from MS images (Fig. 2d, *R*² = 0.38).

### Variability of the multispectral indices

Vegetation indices resulting both from functional and architectural plant features were computed from MS imaging. All indices (*NDVI*, *GNDVI*, *MCARI2*, and *PRI*, Tables 1 and 2) exhibited a wide range of variability within the core-collection. They displayed a strong effect of the genotype and high heritabilities (0.70 < *H*² < 0.87). High-positive phenotypic correlations were found between *NDVI* and *GNDVI* on one hand (*R*² = 0.52), and between *NDVI* and *MCARI2* on the other hand (*R*² = 0.76, Table 3). By contrast, the correlation between *GNDVI* and *MCARI2* was low, although significant (*R*² = 0.09). Importantly, these correlations were conserved when considering genotypic values, evidencing the consistent behaviors across trees of the same genotype. *PRI* was positively correlated with *GNDVI* (*R*² = 0.40), slightly with *MCARI2* (negative correlation) and not correlated with *NDVI* if genotypic correlations were considered (Table 3). Water deficit generally had a low but significant effect on all the MS imaging indices, except *GNDVI*, with a relative variation between WW and WD of about 10% (Table 2).

**Table 3:** Determination coefficients for the correlations between the indices estimated from multispectral and thermal imaging and the *I*_PL_

	Genotypic values						
**Phenotypic values**	**NDVI**	( + )0.49***	( + )0.85***	( + )0.0022 ^ns^	(−)0.15***	( + )0.19***	( + )0.044**
	( + )0.52***	**GNDVI**	( + )0.15***	( + )0.28***	(−)0.063***	( + )0.12***	( + )0.017 ^ns^
	( + )0.76***	( + )0.090***	**MCARI2**	(−)0.04**	(−)0.13***	( + )0.16***	( + )0.036**
	( + )0.044***	( + )0.40***	(−)0.023***	**PRI**	( + ) 0.032**	( + )0.053***	( + )0.006 ns
	(−)0.12***	(−)0.0044*	(−)0.20***	( + )0.048***	**T**_**surf**_ – **T**_**air**_	( + )0.41***	( + )0.0001 ^ns^
	( + )0.012**	( + )0.029***	( + )0.001 ^ns^	( + )0.023***	( + ) 0.64***	**WDI**	( + )0.026*
	(−)0.0061*	(−)0.029***	( + )0.0009 ^ns^	(−)0.084***	(−) 0.11***	(−)0.11***	**I**_**PL**_

Acronyms as in Table 1. Above the diagonal, correlations calculated on the genotypic values (BLUPs, *n* = 241 genotypes). Below the diagonal, correlations calculated on the individual phenotypic values (*n* = 930 trees). For each correlation, the determination coefficient is indicated together with the sign ( + , positive, −, negative) of the correlation. The correlation coefficients are displayed with their significance level: *p ≤ 0.08; **p ≤ 0.05; ***p ≤ 0.01; ****p ≤ 0.001; ns, non-significant.

### Using fluorescence measurements for estimating genotypic variation of photosynthesis

**Fig. 3:**
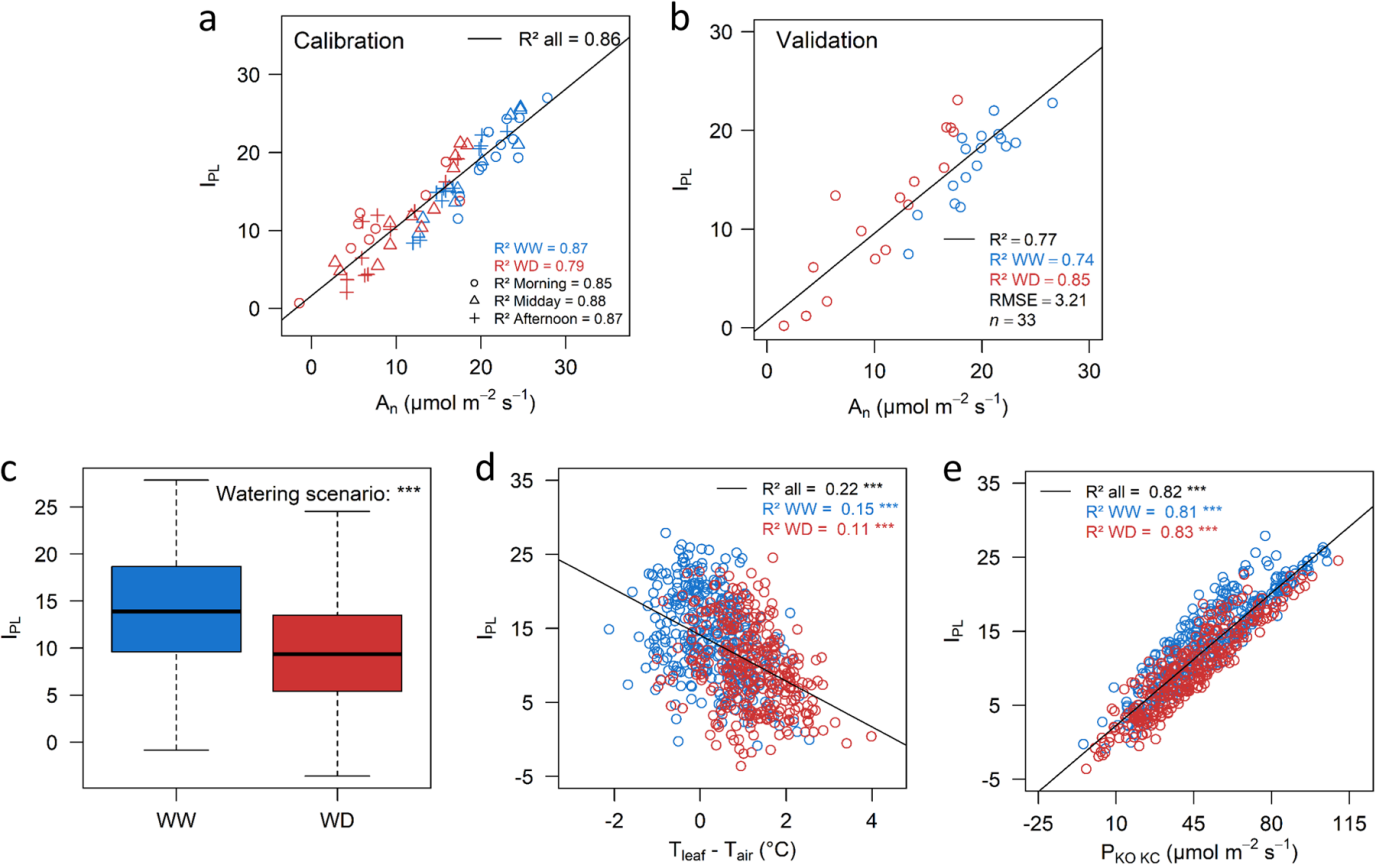
Calibration and validation of the photo-assimilation performance index (*I*_PL_) model, and variability of the *I*_PL_ within the core-collection. **a**, **b** The calibration was obtained by measuring net photosynthesis and fluorescence on 48 trees in a one-day measurement during summer 2017. Calibration model was built using 2/3 of the data (*n* = 93, **a**), and model was then validated on the other 1/3 of the data (*n* = 33, **b**). Data collected at three measurement period (morning, midday, afternoon) are identified with different symbols. In **a** and **b**, calibration and validation are presented for one of the two IRGA devices used (see Supplementary Fig. S4 for the second one). **c** Boxplot representation of the *I*_PL_ measured at high-throughput on the whole core-collection (*n* = 800 trees). The significance of the watering scenario was assessed with a one-way ANOVA. *** significant at *p* < 0.001. **d**, **e** Correlations between the *I*_PL_ and its two components, *T*_leaf_ *–* *T*_air_ (**d**) and *P*_KO/KC_ (**e**) on the whole core-collection. Blue and red points represent trees subjected to well-watered (WW) or water deficit (WD) conditions, respectively. Coefficients of determination and their significances were computed considering either the whole dataset (all), or within each watering scenario independently (WW or WD). Root mean square errors were estimated to assess the consistency of correlations between *A*_n_ and *I*_PL_

In this study, leaf fluorescence measurements were used as a proxy for photosynthesis activity in order to extend the functional phenotyping of the core-collection. The calibration dataset for the *I*_PL_ model covered a wide range of variation for *A*_*n*_ (from 2 to 30 µmol m^−2 ^s^−1^, Fig. 3a and Supplementary Fig. S4a). The device (two IRGAs were used) impacted the range of values recorded for *T*_air_, *T*_leaf_, and *ΦPSII* (Supplementary Table S3), likely due to internal calibration proper to each. Thus, a specific set of parameters was chosen for each device. Both *P*_KO/KC_ and *T*_leaf_ – *T*_air_ (Table 1) had highly significant effects on *I*_PL_ (*p* < 0.0001), confirming the relevance of using both parameters to estimate photosynthesis accurately. Calibrations were robust (*R*² = 0.86 and 0.84, Fig. 3a and Supplementary Fig. S4a) and a unique set of parameter values was relevant whatever the scenario or period (*R*² > 0.79 when splitting the calibration dataset per scenario or daytime). Accuracy was confirmed by the high values of *R*² and root mean square errors (RMSE) obtained in the validation datasets (Fig. 3b and Supplementary Fig. S4b, *R*² = 0.77 and 0.74, respectively, and RMSE = 3.1 and 3.2, respectively). *I*_PL_ values were then estimated at HT on the core-collection, from the calibrated model, and ranged from 0 to 27 among the WW trees and from –5 to 25 for WD ones, with a significant effect of the genotype (Table 2). WD decreased *I*_PL_ values by 30% on average as compared to WW conditions (Table 2 and Fig. 3c). Its variation within the core-collection was mostly driven by variability in *P*_KO/KC_ (Fig. 3e, *R*² = 0.82), which combines electron transport rate exiting from PSII (*J*_PSII_), in turn dependent on the absorbed photosynthetically active radiation and effective PSII efficiency, and, to a lesser extent, by variability in *T*_leaf_ *–* *T*_air_ (Fig. 3d, *R*² = 0.22), itself related to the degree of stomatal closure. Variations in air VPD along the measurement duration also significantly affected *I*_PL_ values (Supplementary Fig. S5). We thus integrated these undesired effects (device, VPD) into the mixed-models used for BLUPs and heritability calculation (Supplementary Table S2). This yielded medium heritability for *I*_PL_ (*H*² = 0.43, Table 2). Low correlation coefficients, although sometimes significant due to the large number of individuals, were found between the *I*_PL_ and the vegetation indices derived from MS imaging (Table 3).

### High-throughput characterization of whole-plant response to water deficit

**Fig. 4:**
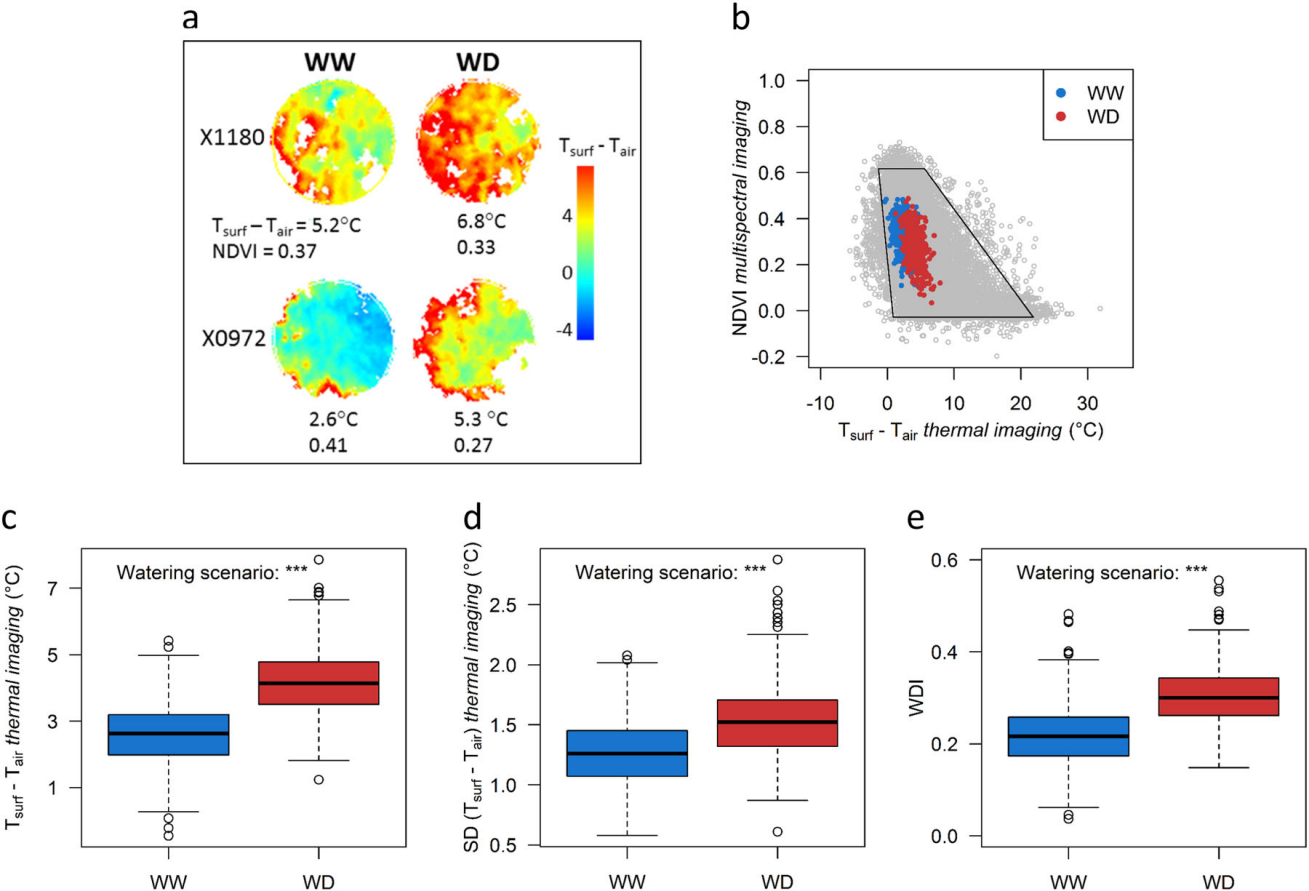
Impact of water deficit on the water deficit index (*WDI*) and the surface-to-air temperature difference (*T*_surf_ *–* *T*_air_). **a** Thermal pixel array within the buffer zone estimated from thermal infrared (TIR) imaging on four trees belonging to two genotypes (X1180 and X0972) under well-watered (WW) and water deficit (WD) conditions on 27^th^ July. Corresponding mean *T*_surf_ *–* *T*_air_ and *NDVI* values are indicated below each tree. **b** Relationship between *NDVI* and *T*_surf_ *–* *T*_air_. Gray points represent all the pixels, including soil, weed, and trees, whereas red and blue points are the mean values of *T*_surf_ – *T*_air_ and *NDVI* for each individual tree (blue: WW trees, red: WD trees). Solid lines represent the trapezoid shape used for computing *WDI*. Extremities of the trapezoid represent “well-watered vegetation” (top left), “water-stressed vegetation” (top right), “satured bare soil” (bottom left) and “dry bare soil” (bottom right) conditions. **c**–**e** Boxplot representations on the whole core-collection of the mean *T*_surf_ *–* *T*_air_ values (**c**), the standard deviation of *T*_surf_ *–* *T*_air_ within the canopy (**d**), and *WDI* (**e**) depending on watering scenarios. In **c**–**e** data are phenotypic values (*n* *=* 930), corrected for fixed effects of date and daytime period. The significance of the watering scenario was assessed with a one-way ANOVA. ***Significant at *p* < 0.001

Our experimental design allowed to further investigate the response of genotypes to soil water deficit as two plants per genotype were subjected to soil water deficit conditions. Whole-plant sensitivity to water deficit was analyzed with two main variables computed from airborne images: *T*_surf _– *T*_air_ and *WDI* (Fig. 4a), over two consecutive dates (27^th^ July at midday and 28^th^ July morning). The acquisition date had a highly significant effect on both variables, with lower values on 28^th^ July (mean *T*_surf_ *–* *T*_air_ = 3.07 and 0.64 °C, and *WDI* = 0.27 and 0.22 for all the trees in 27^th^ July and 28^th^ July, respectively). The lower values on 28^th^ July were likely due to the daytime period differing between both dates, with lower temperature and VPD (Supplementary Fig. S6) in the morning conditions that led to higher stomatal conductance and transpiration rate. Moreover, as image acquisition extended over >45 min with three different elevations for each flight date, some variations also existed within each day of measurement (Supplementary Fig. S6). Despite changes in absolute values, correlations between data collected on the two dates and corrected based on the fixed effects of day and flight within the day remained significant although with medium correlation coefficients (Supplementary Fig. S7).

On 27^th^ July, the four temperatures used for estimating the trapezoid relationship between *NDVI* and *T*_surf_ – *T*_air_ were, respectively, equal to 0.87, −1.34, 21.86, and 5.6 °C for saturated bare soil, well-watered vegetation, dry bare soil, and water-stressed vegetation, respectively (Fig. 4b). Consistently with the observed *T*_surf_ –*T*_air_, these temperatures were lower on 28^th^ July (Supplementary Fig. S8). As expected, whatever the date, *T*_surf_ *–T*_air_ and *WDI* (Fig. 4c, e and Supplementary Fig. S8b, c) increased under water deficit conditions. Moreover, large variations were observed for WD trees with a genotypic coefficient of variation equal to 0.41 and 0.32 under WD conditions for *T*_surf_ *–T*_air_ and *WDI,* respectively. *T*_surf_ –*T*_air_ was more heterogeneous within tree canopy under WD as compared to WW conditions, as shown by the higher standard deviation computed from all vegetation pixels of each tree (Fig. 4d and Supplementary Fig. S8d). Mixed-effect models accounting for scenario, date and elevation effects within each day were used for *WDI* and *T*_surf_ *–T*_air_ on the dataset combining the 2 days of measurements (Supplementary Table S2), yielding high heritabilities for both traits (Table 2).

### Assessments of high-throughput phenotyping methods for functional traits

**Fig. 5:**
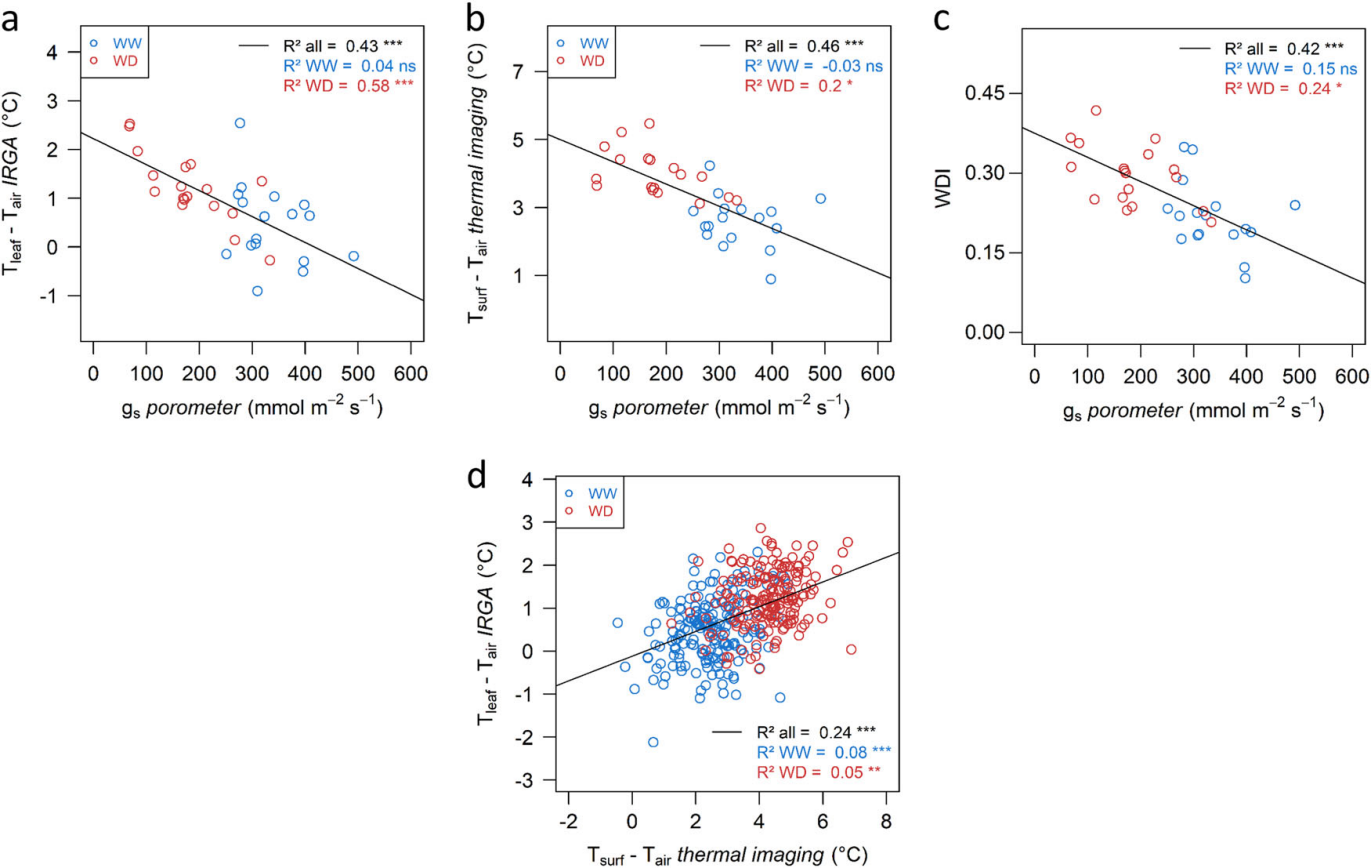
Assessment of high-throughput methods for computing leaf or surface temperatures. **a** Correlation between leaf-to-air temperature difference (*T*_leaf_ *–* *T*_air_) measured with the gas-exchange analyzer during the *I*_PL_ measurement, and stomatal conductance (*g*_s_*)* measured with the porometer. **b** Correlation between the mean value of surface-to-air temperature difference (*T*_surf_ – *T*_air_) obtained by thermal infrared imaging (TIR) and *g*_s_ measured with the porometer. **c** Correlation between water deficit index (*WDI*) and *g*_s_ measured with the porometer. **d** Correlation between *T*_leaf_ – *T*_air_ measured with the gas-exchange analyzer during the *I*_PL_ measurement, and the mean value of *T*_surf_ – *T*_air_ obtained by TIR imaging. In **a**–**c**, *n* = 40 trees; in **d**, *n* *=* 400 trees. All measurements were performed on the same day. *g*_s_ and *T*_leaf_ – *T*_air_ were measured, respectively, on three and one single leaf per tree. Mean *T*_surf_ – *T*_air_ was calculated overall the pixels of each tree

The relevance of HT measurements of functional traits was assessed by comparing *T*_surf_ –*T*_air_ derived from imaging to low-throughput measurements with the IRGA of these variables and to stomatal conductance values. We thus explored the relation between *g*_s_ and the leaf-to-air temperature difference considering the latter at different scales: (i) at the canopy level (mean value of *T*_surf_ –*T*_air_ derived from imaging), and (ii) at the leaf level (*T*_leaf_ –*T*_air_ measured with the IRGA during the *I*_PL_ measurements, on one of every three leaves per tree measured with the porometer). Significant correlations between *T*_surf_ –*T*_air_ (respectively, *T*_leaf_ –*T*_air_) or *WDI* and *g*_s_ were observed when considering the whole dataset (WW and WD) or the WD plants only (Fig. 5a, c). Nevertheless, these correlations were no longer significant for the WW trees. *T*_leaf_ –*T*_air_ and *T*_surf_ –*T*_air_ were significantly correlated (Fig. 5d) but this correlation was mostly driven by the contrast between WW and WD trees, whereas it was no longer significant to discriminate the individuals within each watering regime separately. Vegetation temperature was significantly lower when measured with the IRGA as compared to canopy imaging even though these temperatures were measured at the same time of the day (Fig. 5d). For most of the genotypes, the temperature measured with the IRGA ranked into the first quartile of the pixel canopy temperature estimated from airborne imagery (Supplementary Figs.  S9 and 10).

### Identification of genotype classes based on architectural and functional traits

**Fig. 6:**
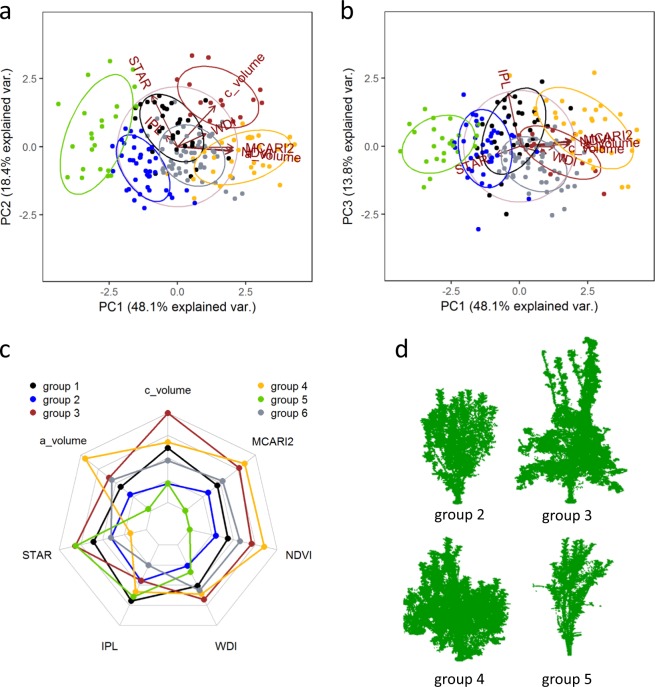
Results of the multivariate analyses performed on the genetic values of the architectural and functional traits on the whole population. **a**, **b** Projections of individuals and variables on the three first axes of the principal component (PC) analysis. Groups represent the six groups identified using a Hierarchical Ascendant Classification. **c** Radar plot of the mean trait values computed using scale variables for the six groups. d T-LiDAR point clouds of 4 trees representative of groups 2, 3, 4 and 5

We performed a PCA analysis on the BLUPs estimated on the whole core-collection with mixed-effect models including, when significant, the watering scenario as fixed-effect to explore the quantitative relationships between variables and similarities between genotypes. Seven variables were chosen to encompass traits (i) related to plant architecture (*c_volume*, *a_volume*), (ii) combining plant architecture and functioning (*NDVI*, *MCARI2*, *STAR*), (iii) accounting for the limitation in leaf functioning due to soil water content (*WDI*) and (iv) representing leaf photosynthesis (*I*_PL_). The first two axes explained >66% of the variance and the first four axes >94% (46.1%, 18.4%, 15.2%, 14.6% for axes 1, 2, 3, and 4, respectively, Fig. 6a, b and Supplementary Fig. S11). The variability in the population was first explained by a combination of variables related to the size of the tree (*a_volume*, *NDVI*, *MCARI2*, PCA’s axis 1) and second by *STAR*, and to a lower extent *c_volume* (PCA’s axis 2). The third and fourth dimensions were almost fully explained by one variable, only (*I*_PL_ or *WDI*, respectively). A hierarchical clustering (Fig. 6c, d) performed on the same variables as those used for PCA allowed the identification of six groups of genotypes. Consistently with the results of the PCA, the population was first separated into two parts depending on variables related to tree size with groups 2 and 5 displaying lowest values. Groups of large and small plants then differed depending on *STAR* values (strongly associated with the ratio of *a_volume* to *c_volume*) with groups 4 and 2 displaying low values, for small and large plants, respectively (Table 4). Among groups of large plants with high *STAR* values, group 6 had the lowest *I*_PL_ values and group 2 was the least sensitive to water stress (low *WDI* value). This clustering analysis showed independence between architectural and functional traits. Indeed, many combinations of plant size and *I*_PL_ values could be observed (e.g., group 5: small plants, high *I*_PL_; group 3: large plants, low *I*_PL_; group 2: small plants, low *I*_PL_; group 4: large plants, medium *I*_PL_).

**Table 4:** Mean values per group for the variables used for clustering analysis

Groups	Number of genotypes	*c_volume*	*a_volume*	*STAR*	*I*_PL_	*WDI*	*NDVI*	*MCARI2*
1	32	3.34^b^	0.76^c^	0.77^a^	12.7^a^	0.24^a,b^	0.31^c^	0.48^b^
2	39	2.21^d^	0.68^d^	0.71^b^	11.7^b^	0.22^c^	0.29^d^	0.44^c^
3	15	4.45^a^	0.87^b^	0.83^a^	11.8^b,c^	0.26^a^	0.35^a,b^	0.56^a^
4	32	3.52^b^	1.08^a^	0.64^c^	12.3^a,b^	0.25^a^	0.37^a^	0.58^a^
5	24	2.21^d^	0.52^e^	0.83^a^	12.5^a,b^	0.23^b,c^	0.25^c^	0.36^d^
6	52	2.94^c^	0.84^b^	0.71^b^	10.9^c^	0.25^a^	0.33^b^	0.50^b^
Group effect	***	***	***	***	***	***	***	

The significance of the group effect was tested with a one-way ANOVA followed by a Tukey’s HSD test for pairwise comparison. ***significant at *p* < 0.001. Values followed by different letters are significantly different at *p* < 0.05

## Discussion

### An original combination of high-throughput tools to phenotype thousands of trees under contrasted watering regimes

HT phenotyping methods were combined to study the genotypic variability of variables related to plant architecture (*a_volume, c_volume, c*_i_*, STAR*), functioning (*I*_PL_), sensitivity to soil water deficit (*WDI, T*_surf_ – *T*_air_) and “mixed” traits accounting both for architectural and functional characteristics (*NDVI*, *GNDVI*, *MCARI2*). This study thus extends the domain of application of new HT methods, up to now mostly deployed on annual species^18^.

To assess the accuracy of these methods, the traits measured at HT were compared to *in planta* measurements, or to other traits acquired at HT with an alternative method. For architectural variables, the significant correlations between *a_volume*, *TLA, TCSA*, *pix_num,* and *NDVI* confirm the relevance of using T-LiDAR or airborne imagery for characterizing whole-tree development. Nevertheless, correlations between T-LiDAR variables and *NDVI* were quite low, likely due to the nature of *NDVI*, which also accounts for features not directly associated with plant architecture (i.e., chlorophyll content). Architectural variables were complemented with *STAR* estimations. *STAR* was negatively correlated with *a*_*volume* and *c*_i_. This first suggests that plants with large leaf area have lower radiation absorption  per unit leaf area, probably due to leaf over-lapping, as previously observed^36^, and second, that *c*_i_ is a good indicator of foliage 3D distribution within the canopy. Interestingly, large genotypic variability was also found among genotypes for *GNDVI* and *MCARI2*, which both depend on leaf area index and chlorophyll concentration^24,25^. *PRI*, which is less dependent on tree architecture but closely associated with radiation-use efficiency, also displayed large variability. Overall, our results underline the relevance of using a 6-channel multispectral acquisition in combination with thermal infrared imagery, which allowed a high spatial resolution and proved to be cost-effective^37^. A technical alternative could rely on hyperspectral acquisition, whose non a-priori approach and high spectral resolution is tempting but would imply lower spatial resolution and more challenging data analysis. Subsequently, in order to estimate photosynthesis at the leaf level, we computed a semi-empirical parameter (*I*_PL_) estimated using a calibration-validation procedure, which revealed the accuracy of this indicator independently of climate or watering scenario. While it was proposed that the model parameters were species specific^17^, we show that they also vary with the device used, hence the importance of running calibration measurements for any new experiment.

### Relevant design and analytical methods allow detecting strong genotypic variability in response to water deficit

Experimental design and timing of measurements are critical to ensure successful HT characterization. In our case, due to local regulation, the drone flight had to be carried out at reduced altitude, thus requiring several successive elevations to cover the entire field. This results in variations of environmental conditions with *T*_surf_ following the variations of *T*_air_ and VPD. By repeating measurements over 2 consecutive days at two distinct periods, we were able to compare the effect of external conditions. Measurements centered on solar midday offered more stabilized conditions. Similarly, for the leaf fluorescence measurements, we made sure to perform measurements under saturating solar radiation conditions to avoid any pitfalls frequently reported as fluorescence measurements are dependent on the ambient light levels^13^. Importantly, the use of mixed-effect models was crucial to get rid of the environmental, uncontrolled variability (e.g., fixed effects of elevation for the airborne imagery-derived indices; of the VPD for the *I*_PL_).

With this approach, we were thus able to evidence strong genotypic effects on all the studied variables, most of them displaying high heritabilities (*H*² > 0.6). Whereas strong genotypic effects on indices derived from airborne imagery were similarly found on annual crops^38^, such high values were not frequently reported on woody crops (e.g., no effect of the genotype from TIR imagery on a poplar biparental population^39^). For architectural traits, heritabilities were similar to or even higher than those obtained in previous studies in field on biparental population^7^ or on the same core-collection in controlled conditions^4^, thus confirming the strong genetic determinism of architectural traits.

Marked genotypic differences were observed between the effects of the watering regimes on the architectural and functional variables. Functional traits revealed average reductions by >30% in *I*_PL_ and to a lesser extent in *PRI*. Leaf temperature strongly increased under water deficit (around + 2 °C) probably due to stomatal closure under these conditions. Such responses are consistent with rapid changes in leaf homeostasis upon soil drying, likely due to hormonal or hydraulic regulations^40^. On the other hand, vegetative and architectural traits (e.g., *a_volume*, *NDVI*) only showed a slight, not always significant reduction in WD as compared to WW trees. Indeed, these integrative traits were built over the whole 4-years tree life, while the WD condition was only established during one month in 2016 and 2017. Besides, in these 2 years, the WD occurred in July, when most of the vegetative development was already achieved.

### On the consistency and discrepancies between scales of analysis

The scale of analysis (from the leaf to the whole-plant) is a recurrent matter of debate for the study of functional traits. Complementarity of our approaches allows tackling this question within the apple core-collection. “Ground-truthing” measurements (e.g., stomatal conductance by porometry) are often conducted in parallel to airborne image acquisitions and confronted to TIR or MS indices. This results in a profusion of studies reporting a wide range of correlations between canopy indices and *in planta* measurements (e.g., *R*² between *g*_s_ and *T*_surf_ – *T*_air_ ranging from 0.27 to 0.92 in several woody crops^41,42^). In our study, the simultaneous measurement of *T*_leaf_ – *T*_air_ (IRGA) and of *T*_surf_ – *T*_air_ (TIR imaging) reveals that a well exposed, well developed leaf typically chosen for gas-exchange measurements generally, but not systematically, falls within the first quartile of values reported from zenithal images on the same tree (Supplementary Figs. S9 and 10 and Fig. 5d). Consistently, the correlation found between *T*_surf_ – *T*_air_ and *g*_s_ was significant with a medium *R*² (0.43) mostly driven by the contrast between watering scenarios rather than genotypes (Fig. 5a), consistent with other reports^39^. Another example is the low correlation found between the *I*_PL_ measured on single leaves and the *PRI* associated with leaf fluorescence but also known to be highly affected by factors such as canopy structure, viewing and illumination geometry effects, and background^43^. As leaves within a tree strongly differ in terms of size, age, position or nitrogen content, care should thus be taken when confronting scales and thinking in terms of comparison should be preferred rather than in terms of validation. Upscaling to the canopy level and assessing the sample heterogeneity, as authorized by imagery techniques, might be crucial to extract relevant information on tree functioning.

### Toward genetic analyses of tree performances in a genotype × environment interaction context

Based on the BLUPs extracted for each trait, we were able to identify six distinct genotypic classes. These results demonstrate the absence of unique relationships between tree functioning, size and architecture, suggesting independent genetic controls. This result also suggests sink activity regulation or other developmental controls (transcriptome, hormones) as drivers of plant growth and phenotype construction^44^. Moreover, the large variations in tree crop loads in the core-collection could have hidden a relationship between architectural and functional traits due to feedback inhibition of photosynthesis under low crop load conditions^45^.

This work will benefit from recent advances in genotyping and the availability of a high-density map for the apple core-collection^46^ to explore the genetic bases of trait variations, using genome-wide association studies. Whereas classical QTL analyses led on fruit tree species often tackle a unique type of traits (e.g., architecture^7^; phenology^47^), our work will offer the opportunity to simultaneously decipher the genetic control of architectural and functional traits. The wide genetic background offered by the core-collection promises a gain for the power of detection and the genericity of detected QTLs as compared to classical approaches led on biparental populations^48^. The portability of our approach will also allow its deployment over other sites across Europe on the same core-collection^47^ and a unique opportunity to decipher the genotype-by-environment interactions ruling the traits of interest. Our protocol could be deployed throughout the gradual establishment of water deficit in order to compute response curve parameters depending on the genotype. Such an approach will complement the results of this study in which we considered the water stress treatment as a fixed-effect at one date. The robustness of our HT indices also suggest their relevance for studies oriented toward agronomical questions, e.g., orchard management on a few varieties. Nevertheless, their further deployment would require the creation of standardized protocols and common data analysis pipelines accessible to non-expert users. This could be achieved within open-access platforms dedicated to phenotyping data analyses (e.g., OpenAlea^32^). Finally, this study was carried out on fruiting adult trees but did not directly account for the HT evaluation of fruit production variation within the population. A number of imagery methods at HT were proposed to detect fruits within the canopy^49^. Nevertheless, this detection is not straightforward due to the large variability in fruit characteristics (size, color) within such a collection. To overcome this problem, new popular methods for remote sensing data based on deep learning could be used to improve the classification of fruits and leaves within the canopy^50^.

## Supplementary information

Supplemental Material
