# Supplementary material for: Multi-scale high-throughput phenotyping of apple architectural and functional traits in orchard reveals genotypic variability under contrasted watering regimes
Source: Hortic Res. 2019 May 1;6:52. doi: 10.1038/s41438-019-0137-3 (PMC6491481; doi:10.1038/s41438-019-0137-3)
Supplement: Supplementary file 1 — Supplemental Material [file 41438_2019_137_MOESM1_ESM.pdf]

Multi-scale high-throughput phenotyping of architectural and functional traits in field/orchard conditions: application to the genotypic variability in an apple tree core-collection under contrasted watering regimes.

Aude Coupel-Ledru[1+], Benoît Pallas[1+*], Magalie Delalande[1], Frédéric Boudon[1,2], Emma Carrié[1], Sébastien Martinez[1], Jean-Luc Regnard[1], Evelyne Costes[1]

[1]UMR AGAP, Univ Montpellier, CIRAD, INRA, Montpellier SupAgro, 34398 Montpellier Cedex 5, France, [2]CIRAD, 34398 Montpellier Cedex 5.

+ Both authors equally contributed to this study.
*Correspondance: benoit.pallas@inra.fr

Aude Coupel-Ledru

Present address: *University of Bristol, School of Biological Sciences, Life Science Building, 24 Tyndall Avenue, Bristol BS8 1TQ, United Kingdom*

Supplementary Information

Table S1. List and description of indexes computed from multispectral and thermal infrared imaging.

Table S2. Mixed-effect models selected for the extraction of BLUPs of genetic values.

Table S3. Values of chlorophyll fluorescence related parameters for the two IRGA devices.

Figure S1. Evolution of soil water potential during the experiment.

Figure S2. Zenithal image of trees in the field.

Figure S3. Correlations between T-LiDAR, imaging data and plant measurements.

Figure S4. Calibration and validation of the I_{PL} model on the second IRGA device.

Figure S5. Effect of the air VPD on the measurements of I_{PL}.

Figure S6. Evolution of the meteorological variables during the drone flights and temporal variation in surface temperature.

Figure S7. Correlation between imaging data collected on July 27th and 28th.

Figure S8. Overview of the impact of water stress on imaging data on July 28th.

Figure S9. Comparison between the distribution of surface temperatures within the canopy pixels (thermal infrared imaging), and leaf temperatures measured on a single fully developed leaf (IRGA), for 8 genotypes.

Figure S10. Comparison between the distribution of surface temperatures within the canopy pixels (thermal infrared imaging), and leaf temperatures measured on a single fully developed leaf (IRGA), for the whole core-collection.

Figure S11. Additional results from the principal component analysis performed on architectural and functional variables.

Table S1. Description of indexes computed from multi-spectral and thermal infrared imaging on the core-collection.

Index	Description	Equation	Region	Reference
NDVI	Normalized Difference Vegetation Index	$\frac{R850 - R675}{R850 + R675}$	0.70m radius buffer	Rouse *et al.*, 1974
GNDVI	Green NDVI	$\frac{R850 - R570}{R850 + R570}$	0.70m radius buffer	Gitelson *et al.*, 1996
MCARI2	Modified Chlorophyll Absorption Ratio Index Improved	$\frac{1.5 \times [2.5 \times (R850 - R675) - 1.3 \times (R850 - R570)]}{\sqrt[2]{(2 \times R850 + 1)^2 - (6 \times R850 - 5 \times \sqrt{R675}) - 0.5}}$	0.70m radius buffer	Haboudane *et al.*, 2004
PRI	Photochemical Reflectance Index	$\frac{R570 - R530}{R570 + R530}$	0.70m radius buffer	Gamon *et al.*, 1992
T_{surf} - T_{air}	Canopy to air difference temperature	$T_{surf} - T_{air}$	Vegetation pixels excluding soil and mixed pixels	Jones, 1999
WDI	Water Deficit Index	$WDI_i = \frac{(T_{surf} - T_{air})_i - (T_{surf} - T_{air})_{WW}}{(T_{surf} - T_{air})_{WW} - (T_{surf} - T_{air})_{WD}}$	All pixels of the field	Moran *et al.*, 1994 Virlet *et al.*, 2014

Rxxx, the reflectance for a *xxx* nm wavelength.

For *WDI*: $(T_{surf} - T_{air})_i$, the average values of $(T_{surf} - T_{air})$ on all the pixels in the area of interest for the tree *i*, and $(T_{surf} - T_{air})_{WW}$ - $(T_{surf} - T_{air})_{WD}$ the hypothetical $(T_{surf} - T_{air})$ values of pixel displaying the same *NDVI* than the considered trees under WW or WD conditions, respectively. In this study the four extremities of the trapezoid shape (well-watered vegetation, water-stressed vegetation, saturated bare soil, and dry bare soil) were estimated on 0.5% of the pixels with the lowest or highest NDVI. All the pixels of this selection were used whatever their origin (trees, grass, soil). This approach allows defining two lines corresponding to the minimal or maximal $T_{surf} - T_{air}$ values depending on *NDVI* variations.

References:

Gamon *et al.* 1992. *Remote Sensing of Environment* **41**: 35–44
Gitelson *et al.* 1996. *Remote Sensing of Environment* **58**: 289–298.
Haboudane *et al.* 2004. *Remote Sensing of Environment* **90**: 337–352
Jones 1999. *Plant, Cell & Environment* **22**: 1043–1055
Moran *et al.* 1994. *Remote Sensing of Environment* **49**: 246–263
Rouse *et al.* 1974. *3rd ERTS Symposium. NASA SP-351, I, 309-317.*
Virlet *et al.* 2014. *Journal of Experimental Botany* **65**: 5429–5442

Table S2. Mixed-effect models selected for the extraction of BLUPs of genetic values. Models selected were those with the lowest Bayesian Information Criterion (BIC), among several mixed models.

Trait	Mixed-model
TCSA	$P_{ij} = \mu + G_i + S_j + e_{ij}$
c_volume	$P_{ij} = \mu + G_i + S_j + e_{ij}$
a_volume	$P_i = \mu + G_i + e_{ij}$
c_i	$P_{ijk} = \mu + G_i + S_j + e_{ij}$
STAR	$P_{ijk} = \mu + G_i + S_j + R_k + e_{ij}$
T_{surf} - T_{air}	$P_{ijk} = \mu + G_i + S_j + D_k + E_k + e_{ij}$
NDVI	$P_{ijk} = \mu + G_i + S_j + D_k + E_k + e_{ij}$
GNDVI	$P_{ijk} = \mu + G_i + S_j + D_k + E_k + e_{ij}$
MCARI2	$P_{ijk} = \mu + G_i + S_j + D_k + e_{ij}$
PRI	$P_{ijk} = \mu + G_i + S_j + D_k + E_k + e_{ij}$
WDI	$P_{ijk} = \mu + G_i + S_j + D_k + e_{ij}$
pix_num	$P_{ijk} = \mu + G_i + S_j + D_k + E_k + e_{ij}$
I_{PL}	$P_{ij} = \mu + G_i + S_j + A_k + V_x + e_{ij}$
T_{leaf} -T_{air}	$P_{ij} = \mu + G_i + S_j + A_k + V_x + e_{ij}$
$P_{KO/KC}$	$P_{ij} = \mu + G_i + S_j + A_k + V_x + e_{ij}$

Trait acronyms as in Table 1.

P is the phenotypic value of genotype, G the random genotypic effect, S the fixed effect of watering scenario, A the fixed effect of the device (IRGA) used, V the fixed effect of the air VPD, R the position of the tree according to the T-LiDAR, D the measurement date and E the elevation within each day of measurements (3 elevations per day), e the residuals. Analyses were performed on the 'multi-scenario' dataset (WW+WD).

For R, three positions were defined (1, 2 and 3) since the T-LiDAR device was positioned every five trees along each row. R=1 means that the T-LiDAR was in front of the tree, R=2 was used when there was one tree between the considered tree and the T-LiDAR, and R=3 for a two tree interval between the tree and T-LiDAR.

Table S3. Comparison, for the two IRGA devices used, of the range of raw values (mean ± SD) recorded for *ΦPSII* and T_{leaf} - T_{air} within the I_{PL} calibration experiment, of the determination coefficients obtained either using a model with one parameter ($P_{KO\ KC}$ or T_{leaf} - T_{air}) or with both parameters, and of the parameters coefficients obtained with the complete model.

Device	*ΦPSII*	T_{leaf} - T_{air} (°C)	R^2 (I_{PL} vs A_n) I_{PL} model with $P_{KO\ KC}$ only	R^2 (I_{PL} vs A_n) I_{PL} model with T_{leaf} - T_{air} only	R^2 (I_{PL} vs A_n), I_{PL} complete model	α complete model	β complete model
LI-COR 1	0.17 ± 0.049	0.55 ± 0.69	0.79	0.65	0.86	0.33	-2.85
LI-COR 2	0.22 ± 0.060	1.31 ± 0.80	0.80	0.70	0.84	0.24	-1.51

Trait Acronyms as in Table 1.

n = ca. 100 measurements for each device. For each device separately, we tested a linear model either only with $P_{KO/KC}$, or only with T_{leaf} - T_{air}, or with both parameters (complete model) to estimate A_n. R^2 displayed are the determination coefficients of the correlation between I_{PL} and A_n within the calibration dataset. *α* is the coefficient of $P_{KO/KC}$ and *β* the coefficient of T_{leaf} - T_{air}.

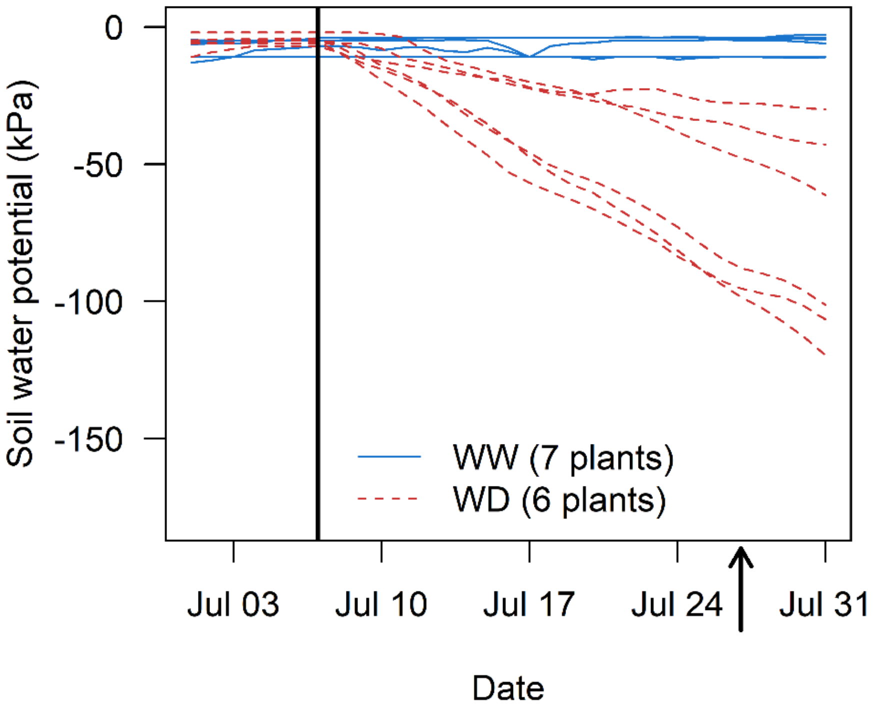

Figure S1. Evolution of soil water potential during the experiment. Soil water potential (Ψ_{soil}) was measured at 60 cm depth by Watermark® probes for the subset of 13 trees (7 well-watered, WW, and 6 water deficit, WD) monitored during summer 2017 in Montpellier. All trees were well-irrigated (2h everyday) until July 5th (black line). The irrigation was then limited to 2h twice a week for the WD trees. The date when the measurements were undertaken (imagery, fluorimetry, porometry) is indicated with a black arrow.

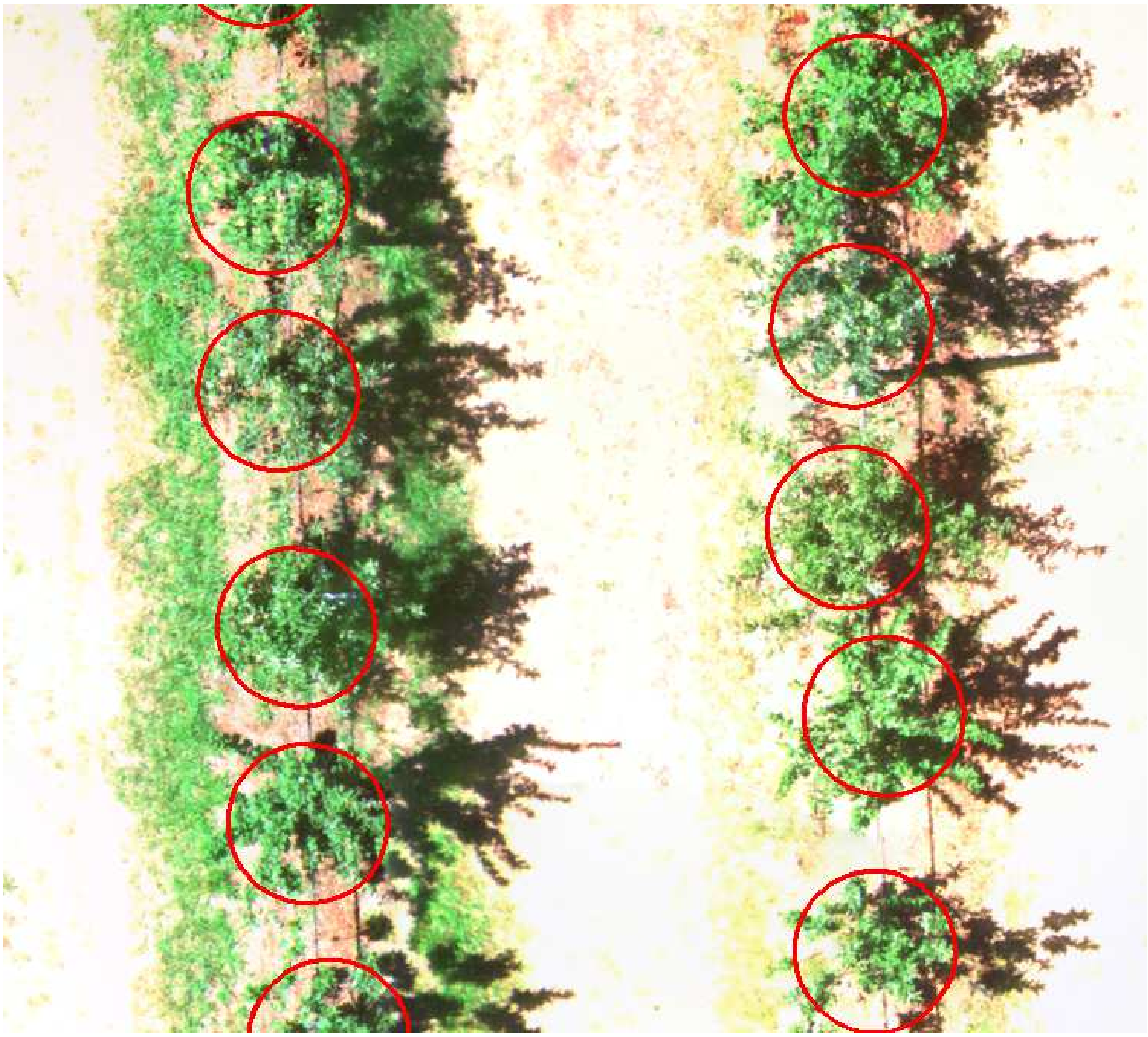

Figure S2. **Zenithal images of trees in the field**. Red circles above each tree represent the zone of interest (radius = 0.70m, buffer zone) on which imaging data (thermal infrared, TIR ; and multispectral, MS) were estimated.

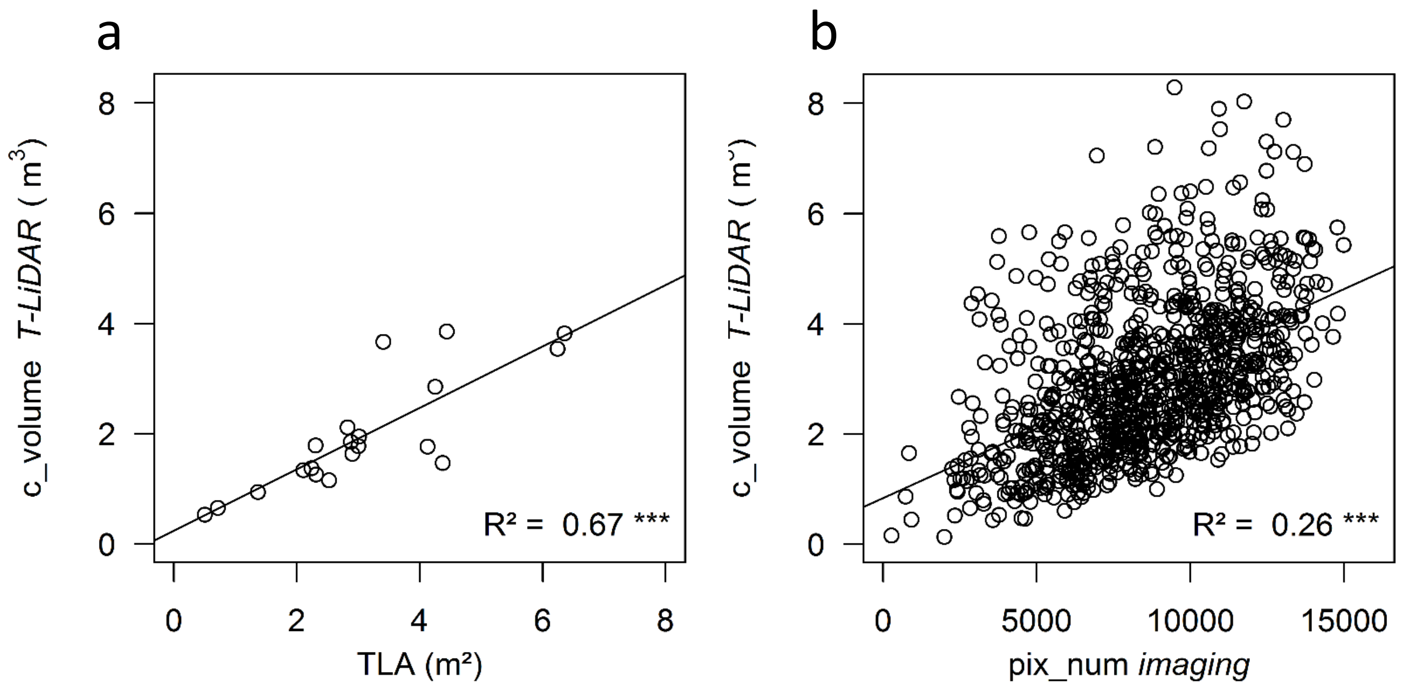

Figure S3. **Correlations between T-LiDAR variables, imaging data and plant measurements**. **(a)** Correlation between convex hull volumes (*c_volume*) computed with T-LiDAR and tree leaf areas (*TLA*) measured on a subset of 20 trees in October 2017. **(b)** Correlation between *c_volume* and pixel numbers (*pix_num*) in the area of interest in the airborne images (buffer zone) on the whole core-collection.

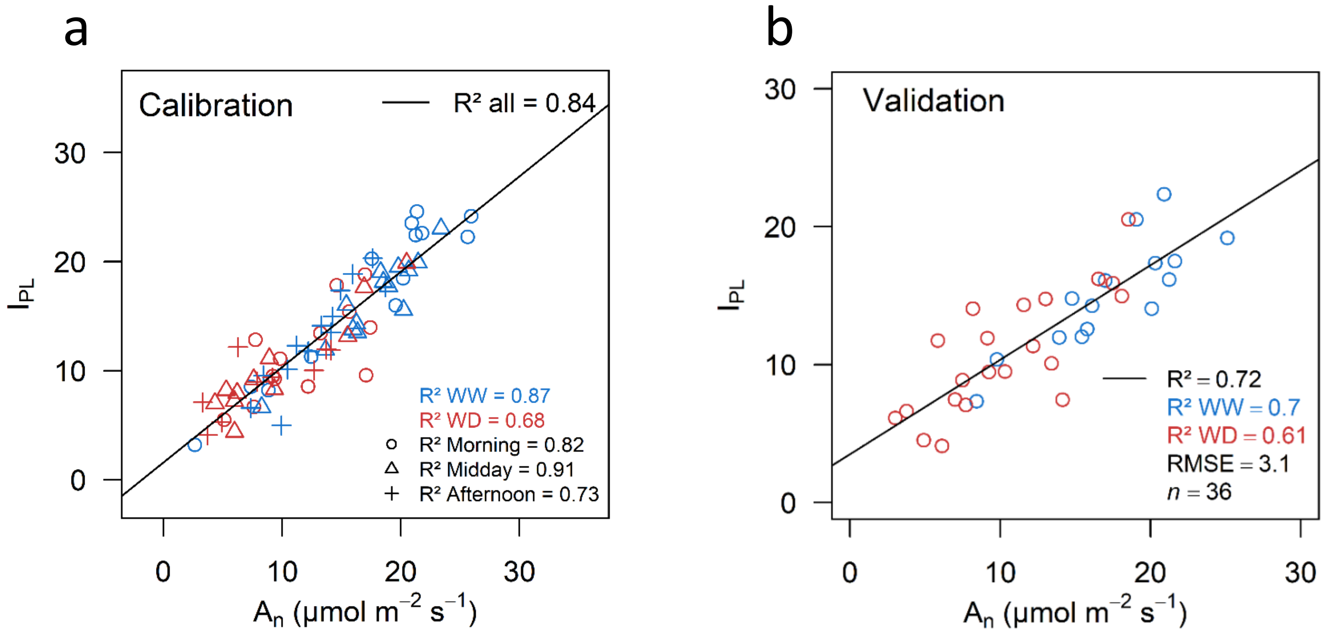

Figure S4. **Calibration and validation of the I_{PL} model on the second IRGA device**. The calibration was obtained by measuring net photosynthesis (A_n) and fluorescence on 24 trees in a one-day measurement campaign during summer 2017. Measurements were repeated at morning, midday, and afternoon. **(a)** Calibration model was built using 2/3 of the data (n = 93), and **(b)** model was then validated on the other 1/3 of the data (n = 33). Data collected at three periods of measurements (morning, midday, afternoon) are identified with different symbols.

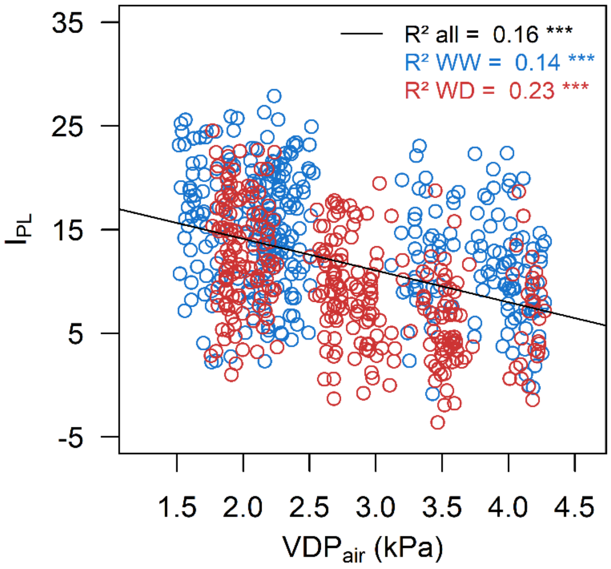

Figure S5. Effect of the air VPD on the measurements of *I_{PL}*. *I_{PL}* measurements were collected on 195 genotypes (2 well-watered (WW) and 2 water deficit (WD) trees per genotype) over two consecutive days (July 27th and 28th) during a maximum period of 3 hours centered on solar midday. The air VPD was simultaneously recorded every 10 seconds by a meteorological station settled within the core-collection.

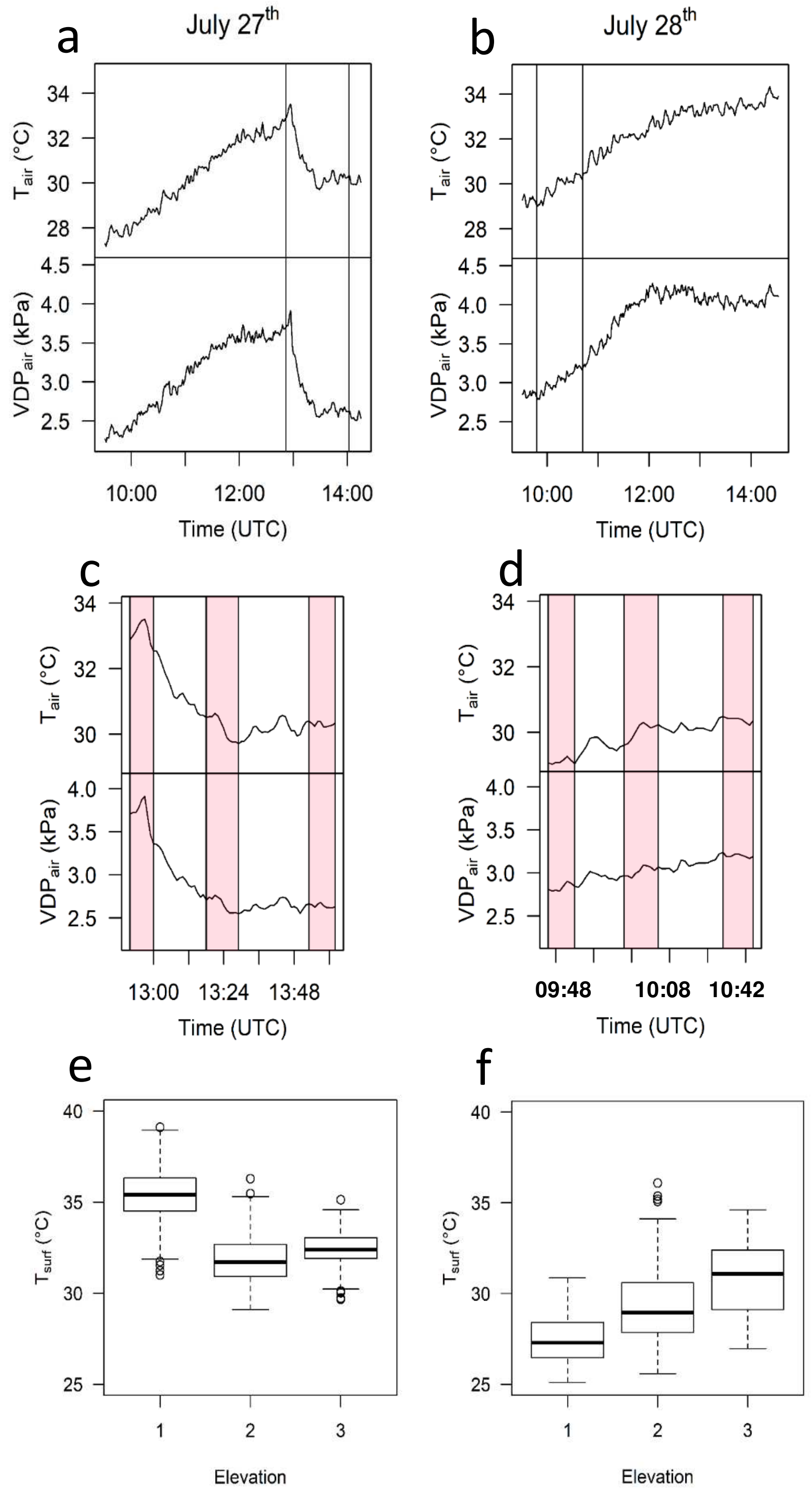

Figure S6. **Evolution of the meteorological variables during the drone flights and temporal variation in surface temperature (*T_{surf}*) on July 27th (a, c, e) and July 28th (b, d, f)**. **(a, b)** Evolution of air temperature (*T_{air}*) and VPD between 9h30 and 15h UTC. The flight window is indicated between vertical bars (time of the first and last image taken) for each date. **(c, d)** Evolution of air temperature and VPD within the flight window specific to each date. Each "flight" was composed of three successive elevations, which are figured by the red rectangles. **(e, f)** Boxplots of the mean *T_{surf}* per elevation.

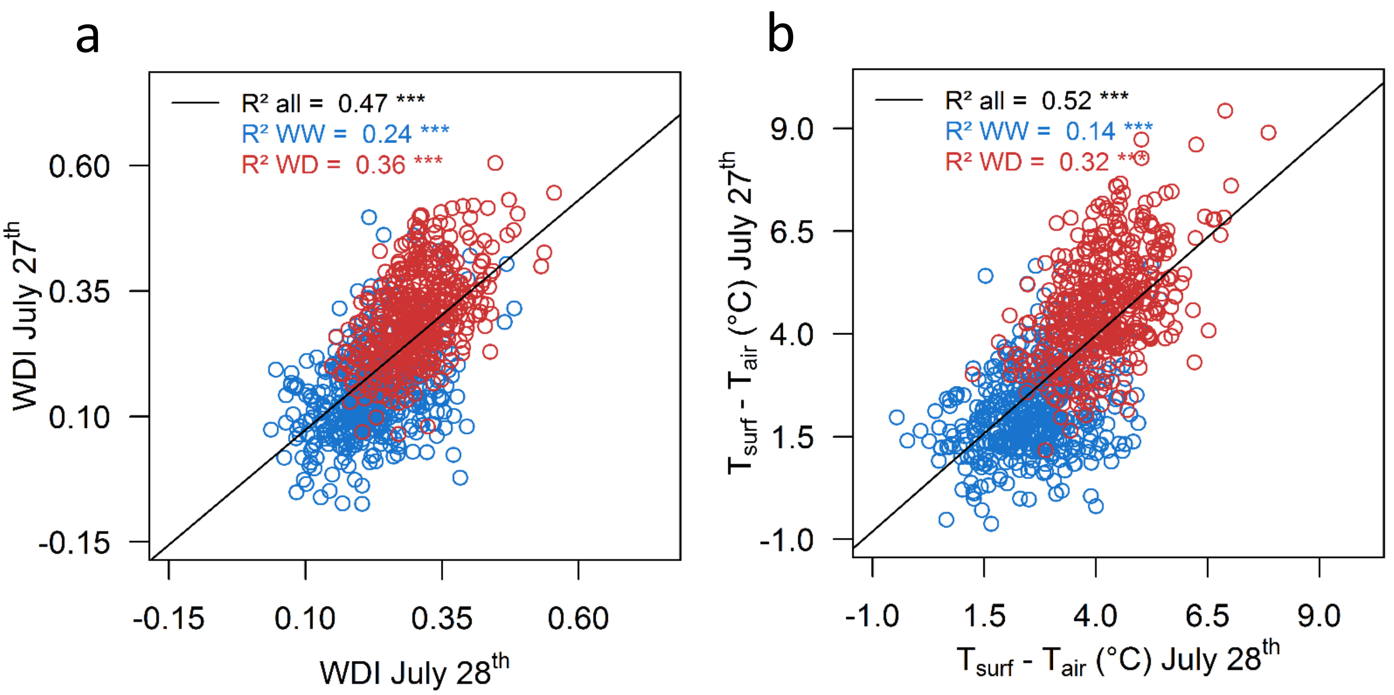

Figure S7. Correlation between imaging data collected on July 27th and 28th. Correlation **(a)** between water deficit index (*WDI*) on both dates and **(b)** between surface-to-air temperature difference (T_{surf} - T_{air}) on both dates. In both panels, data are phenotypic values (n = 930), corrected for fixed effects of date and daytime period. Coefficients of determination and their significances were computed considering either the whole dataset (all), or within each watering scenario independently (WW or WD).

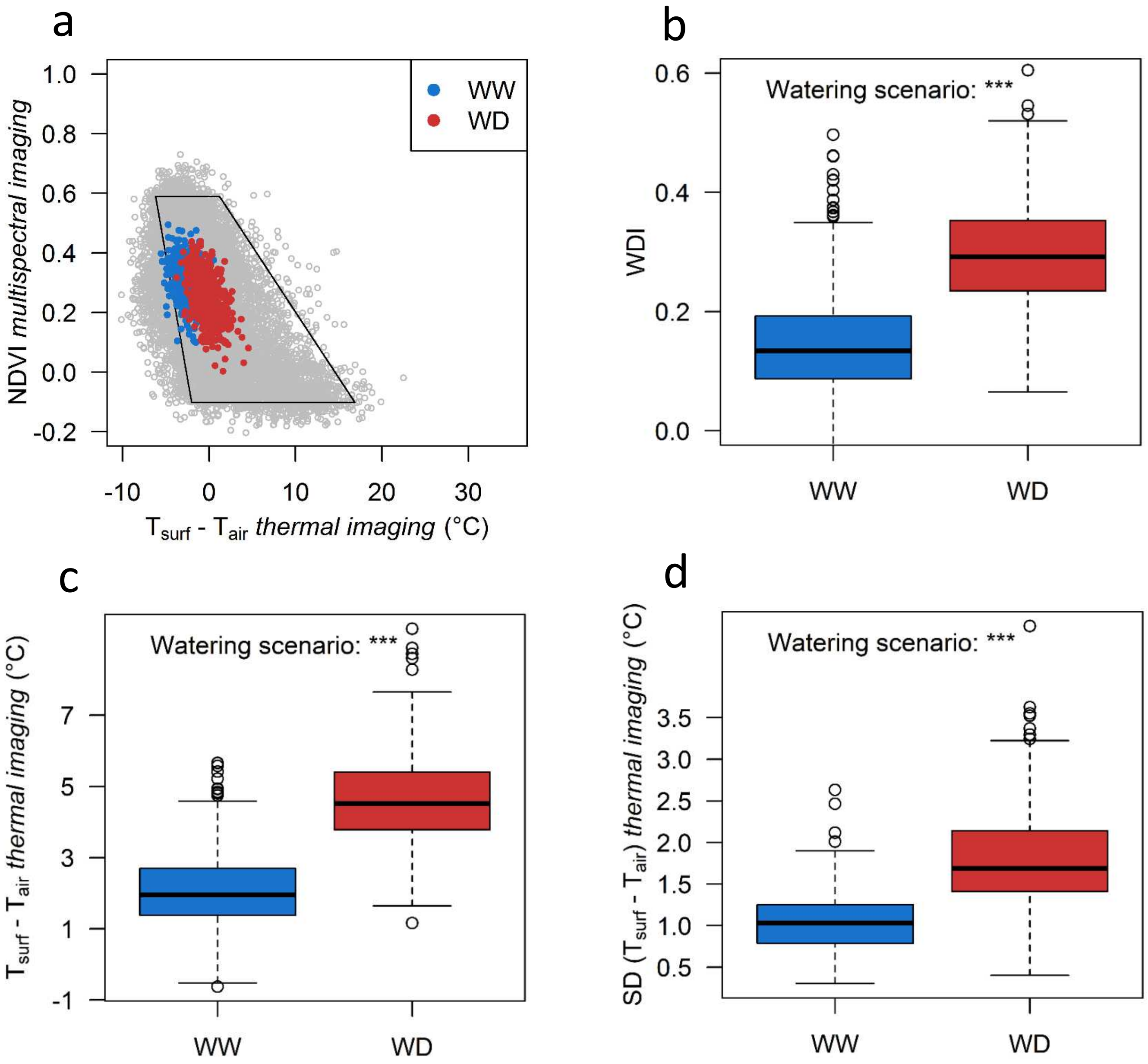

Figure S8. Overview of the impact of water deficit on imaging data on July 28th. **(a)** Relationship between surface-to-air temperature difference (T_{surf} - T_{air}) and *NDVI* from images taken on July 28th (flight time from about 10 to 11am UTC). Grey points represent all the pixels including soil, weed and trees' foliage whereas red and blue points are the mean values of T_{surf} - T_{air} and *NDVI* for each individual tree (blue: WW trees, red: WD trees). Solid lines represent the trapezoid shape used for computing *WDI* (see Table S1). Extremities of the trapezoid represent "well-watered vegetation" (top left), "water-stressed vegetation" (top right), "saturated bare soil" (bottom left) and "dry bare soil" (bottom right) conditions. **(b, c, d)** Boxplot representations on the whole population of the mean *WDI* values **(b),** $T_{surf} – T_{air}$ values **(c)** and the standard deviation of $T_{surf} – T_{air}$ **(d)** depending on watering scenarios, on July 28th. In **(b, c, d)** data are phenotypic values, (n = 930) corrected for fixed effects of date and daytime period. The significance of the watering scenario was assessed with a one-way ANOVA. *** significant at p<0.001.

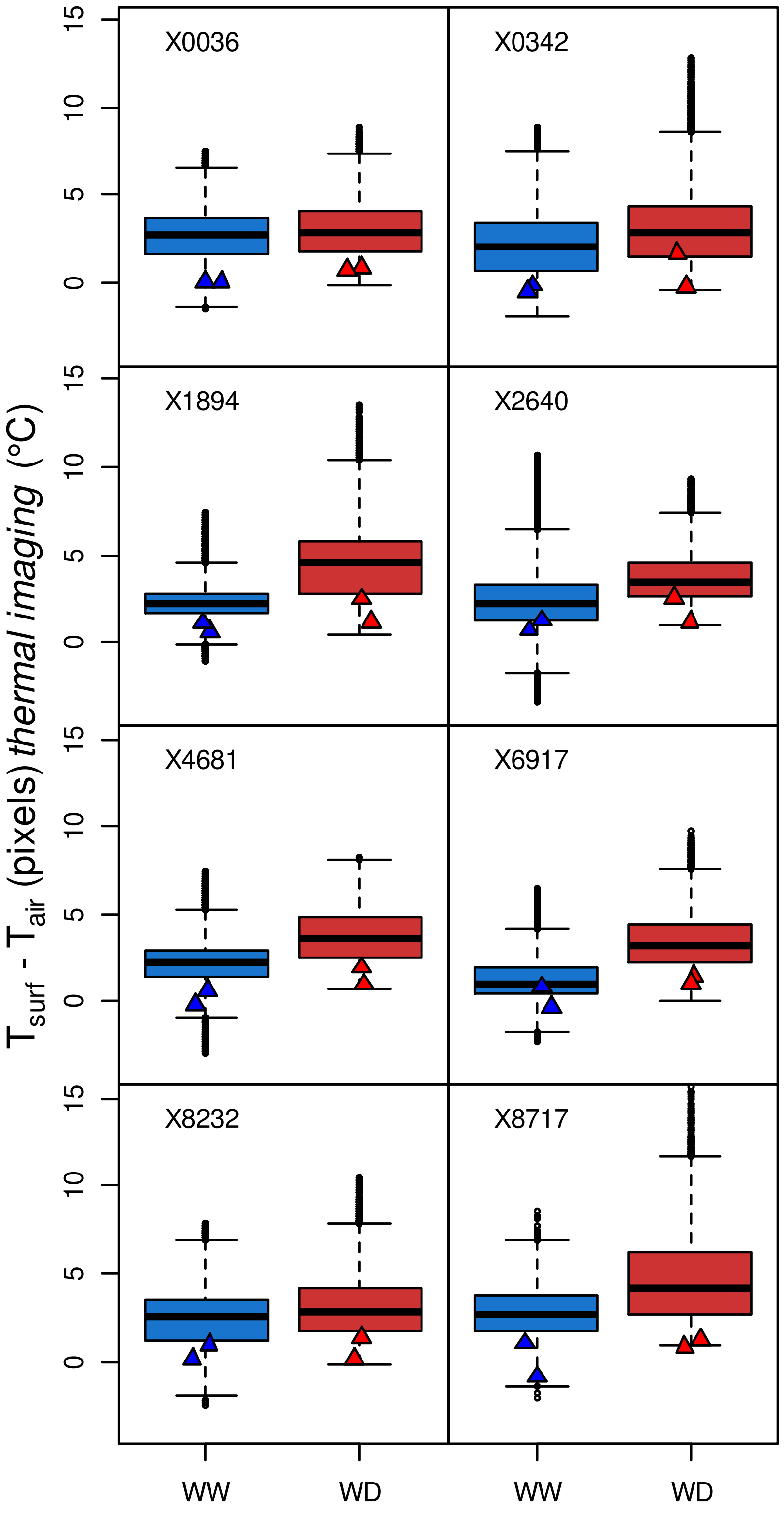

Figure S9. **Comparison between the distribution of surface temperatures within the canopy pixels (thermal infrared imaging), and leaf temperatures measured on fully developed leaves with a leaf gas analyzer, for 8 genotypes**. Boxplot of the surface-to-air temperature difference (T_{surf} - T_{air}) of all canopy pixels, and value within this distribution of leaf-to-air temperature difference (T_{leaf} - T_{air}) measured with the gas-exchange analyzer (triangles). In each boxplot, the values of the two trees for the genotype and scenario considered are represented. The genotype name is indicated in the top-left corner of each panel. Blue and red boxplots correspond to well-watered (WW) and water deficit (WD) trees, respectively.

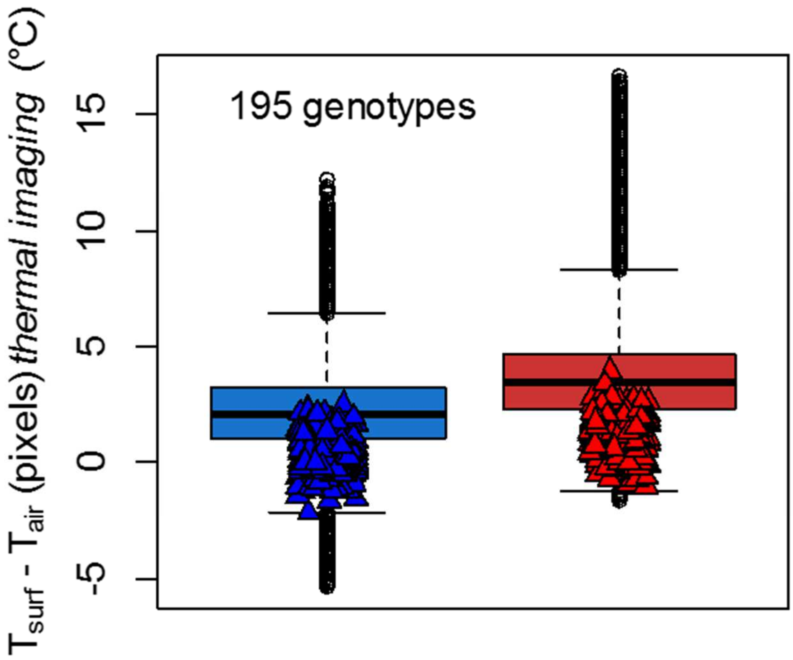

Figure S10. **Comparison between the distribution of surface temperatures within the canopy pixels (thermal infrared imaging), and leaf temperatures measured on fully developed leaves measured with a leaf gas analyzer, for the whole core-collection.** Boxplot of the surface-to-air temperature difference (T_{surf} - T_{air}) of all canopy pixels, and value within this distribution of leaf-to-air temperature difference (T_{leaf} - T_{air}) measured with the gas-exchange analyzer (triangles). Blue and red boxplots correspond to well-watered (WW) and water deficit (WD) trees, respectively. In each boxplot, the values of the two trees for the 195 genotypes and the scenario considered are represented

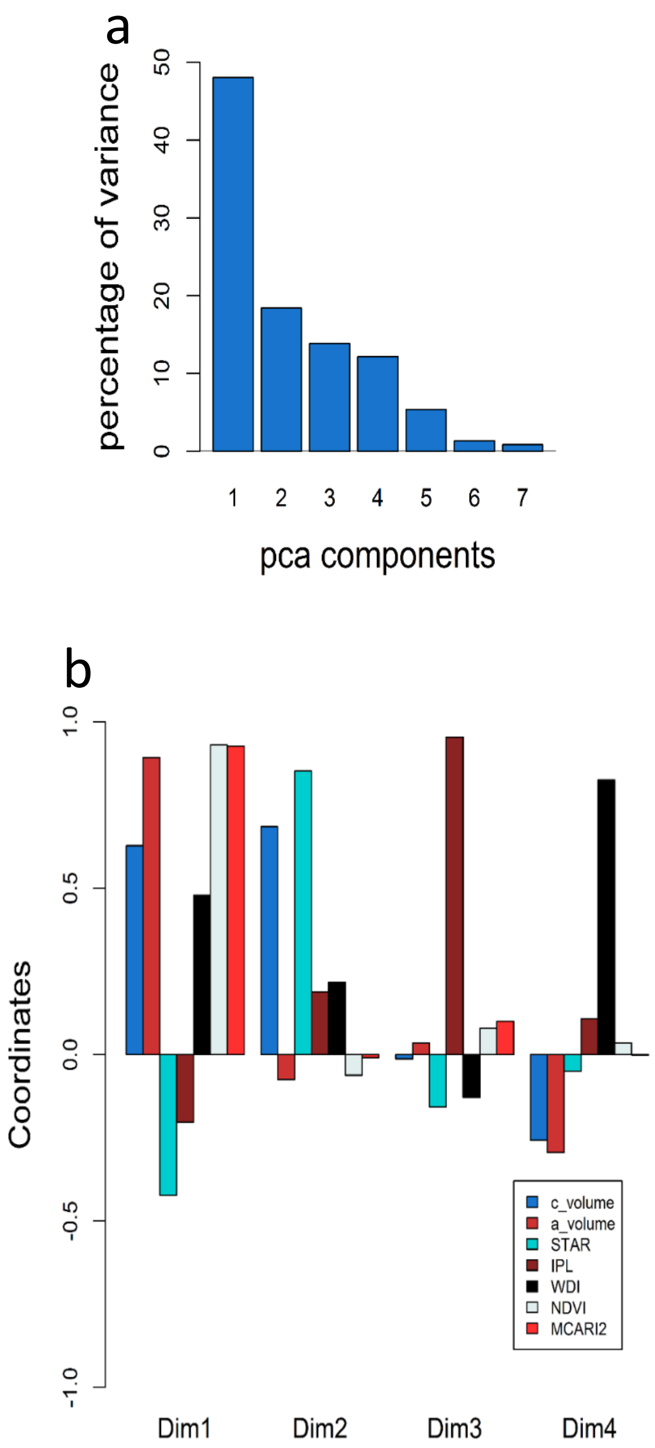

Figure S11. Additional results of the principal component analysis (PCA) performed on architectural and functional variables. **(a)** Percentage of variance explained by each component of the principal component analysis. **(b)** Coordinates of the variables in the four first dimensions of the PCA.
